# NAT10 promotes gastric cancer metastasis via N4-acetylated COL5A1

**DOI:** 10.1038/s41392-021-00489-4

**Published:** 2021-05-03

**Authors:** Yigan Zhang, Yuanxue Jing, Yinxue Wang, Jianming Tang, Xiaoran Zhu, Wei-Lin Jin, Yiqing Wang, Wenzhen Yuan, Xiangkai Li, Xun Li

**Affiliations:** 1 The First Hospital of Lanzhou University, Lanzhou, P.R. China; 2 Key Laboratory for Biological Therapy and Regenerative Medicine Transformation Gansu Province, Lanzhou, P.R. China; 3 The First School of Clinical Medicine, Lanzhou University, Lanzhou, P.R. China; 4 Institute of Cancer Neuroscience, Medical Frontier Innovation Research Center, The First Hospital of Lanzhou University, The First Clinical Medical College of Lanzhou University, Lanzhou, P.R. China; 5 Medical Frontier Innovation Research Center, The First Hospital of Lanzhou University, Lanzhou, P.R. China; 6 School of Life Sciences, Lanzhou University, Lanzhou, P.R. China

**Keywords:** Epigenetics, Gastrointestinal cancer

**Dear Editor**,

Gastric cancer (GC) is among the most prevalent gastrointestinal malignancies. The occurrence of local deep infiltration or distant metastasis in GC is commonly associated with weak treatment and poor prognosis.^1^ Although, N4-Acetylcytidine (ac4C) represents one of the extensive chemical modifications in mRNAs that plays a pivotal role in modulating mRNA stability and the mRNA translation process (Fig. 1b). However, the role of mRNA ac4C modification in disease remains unclear.^2^ As the only known ac4C “writer” protein, NAT10 is thought to have critical effects in tumor metastasis and tumor cell epithelial-to-mesenchymal transition (EMT). Here, we report a novel mechanism of NAT10-mediated mRNA ac4C modification regulating gastric cancer metastasis and EMT.

**Fig. 1:**
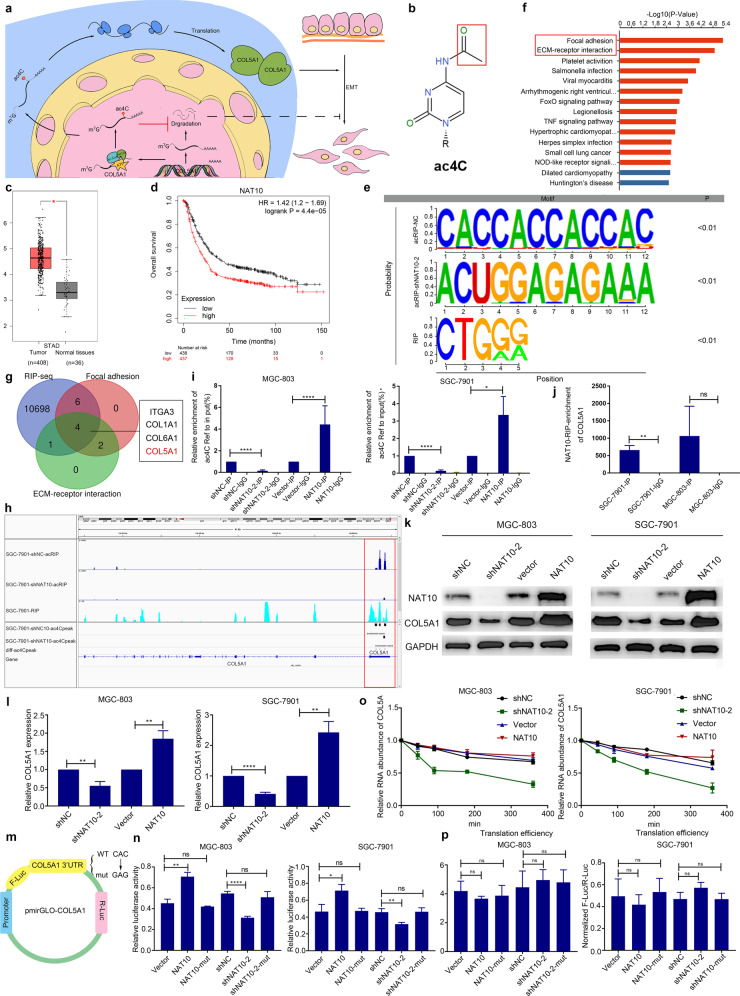
NAT10 promotes gastric cancer progression through mRNA ac4C modification. **a** Graphical summary of this article. **b** The chemical structure of ac4C. **c** The expression of NAT10 in the TCGA STAD dataset. **d** High NAT10 expression was significantly associated with a shorter OS. **e** The ac4C significant consensus sequence motif was identified based on the acRIP-seq and RIP-seq analyses, and the significant consensus sequence of NAT10 bound and interacted. **f** KEGG pathway enrichment analysis and acRIP-seq analysis identified the enriched pathway of ac4C modification down-regulated genes after NAT10 silencing. **g** Overlapping the potential target bound and interacted with NAT10 in focal adhesion and ECM-receptor interaction pathway by the acRIP-seq and RIP-seq analysis. **h** Attenuation of the NAT10 diminishes the ac4C modification genome of COL5A1 mRNA visual result compared using the acRIP-seq (colored in deep blue). The visual genome result of COL5A1 mRNA bound and interacted with NAT10 (colored in wathet blue). **i** The regulatory role of NAT10 on COL5A1 ac4C in SGC-7901 and MGC-803 cells confirmed by the acRIP-qPCR assay. **j** The bound and interacted relationship between NAT10 and COL5A1 mRNA in SGC-7901 and MGC-803 cells confirmed using the RIP-qPCR assay. **k, l** Western blotting and qRT-PCR analyses of COL5A1 in shNAT10 or NAT10 overexpressing MGC-803 and SGC-7901 cells. **m** Wild-type (WT) or mutant COL5A1 cells were transfected with pmirGLO-COL5A1 reporter, respectively. **n** The transcriptional level of wild-type COL5A1, but not the mutation, significantly decreased in the NAT10-knockdown cells, and significantly increased in the NAT10 overexpressing cells. **o** The impression of NAT10 on COL5A1 mRNA stability confirmed by the RNA decay assay. **p** The impression of NAT10 on COL5A1 mRNA translation efficiency confirmed

To explore this issue, we first performed immunohistochemical (IHC) and bioinformatics analysis to evaluate the correlation between the expression levels of NAT10 in GC and clinical information. The results suggested that the expression of NAT10 in GC tissues was upregulated (Fig. 1c), and the expression level of NAT10 was correlated with the AJCC stage and lymph node metastasis (Supplementary Table S2). GC patients with high NAT10 expression also exhibited a significantly poorer OS (overall survival), PPS (post-progression survival), and FP (first progression) than those with low NAT10 expression (Fig. 1d and Supplementary Fig. S1c, d). Western blot analysis and qPCR assays were performed to detect NAT10 expression level in gastric epithelial cells GES-1 and 4 GC cell lines (SGC-7901, BGC-823, MGC-803, and AGS). The results showed that NAT10 expression level was upregulated at the protein level in GC cell lines compared with the gastric epithelial cell lines. However, at the RNA level, NAT10 was only expressed at a significantly higher level in SGC-7901 and MGC-803 cell lines compared with GES-1 cell lines (Supplementary Fig. S1g, h). Based on the results above, we utilized cell wound-healing and transwell matrigel invasion assay to evaluate the invasion and migration ability of MGC-803, BGC-823, SGC-7901, and AGS cells. The result showed that as for invasion and migration, AGS is better than MGC-803, BGC-823 and SGC-7901. However, considering the cell culture medium of AGS is F12, which is different from the other cells, we believe that AGS is not comparable (Supplementary Fig. S11e–h). Next, in vitro and in vivo experiments were conducted to investigate the effect of NAT10 on GC metastasis. In vitro experiments demonstrated that the silencing of *NAT10* significantly impeded the migratory ability of SGC-7901 and MGC-803 cells, while overexpression of *NAT10* enhanced the migratory ability of SGC-7901 and MGC-803 cells (Supplementary Fig. S2a–e). In vivo experiments showed that the downregulation of *NAT10* expression inhibited tumor metastasis of GC cells (Supplementary Fig. S3d, e). To explore the relationship between NAT10 and EMT, WB, qPCR, and immunofluorescence assay were performed. The result demonstrated that *NAT10* was positively correlated with the expression of VIM and MMP2, but CDH1 exhibited no obvious relationship (Supplementary Figs. S4a–d,  S12, and 13). IHC, WB, and qPCR assay using tumor tissues obtained from the subcutaneous tumorigenesis experiment have verified the result (Supplementary Fig. S4e–g). Therefore, NAT10 was suggested to play a promoting role in the EMT process of GC cells.

To explore the regulatory role of NAT10 in EMT and metastasis of GC, acRIP-seq and RIP-seq were performed. The result showed that the ac4C modification downregulated genes were significantly enriched in gene sets involved in the ECM pathway and the cell adhesion pathway after *NAT10* silencing (Fig. 1f). Further analysis of acRIP-seq and RIP-seq data showed that *COL5A1* was a direct target of NAT10-mediated mRNA ac4C modification, and NAT10 regulated ac4C modification on *COL5A1* mRNA 3′UTR through direct interaction (Fig. 1i, j). On the basis of the experiments above, conducting homology analysis did not find the homology of COL5A1 with ITGA3, COL6A1, COL1A1 (Supplementary Fig. S11i, j). The researches before have found COL5A1 a marker of EMTII in tumor cells for it could promote EMT directly.^3,4^

We have known the regulatory mechanism of NAT10 on *COL5A1* mRNA ac4C. However, the effects of this regulation on COL5A1 expression remain unknown. qPCR and WB were used to detect the COL5A1 expression with the change of *NAT10* expression at the cell level. The results showed that knockdown of *NAT10* significantly reduced COL5A1 expression, and overexpression of *NAT10* upregulated the expression of COL5A1(Fig. 1k, l). And the results were also verified by the IHC, WB, qPCR experiments performed on tumor tissues from subcutaneous tumorigenesis experiments (Supplementary Fig. S6g–i). The luciferase report assay also indicated that NAT10 directly regulated the expression of COL5A1 and that this was dependent on the modification of ac4C on *COL5A1* mRNA 3′UTR (Fig. 1m, n). To further assess the effect of *NAT10*-mediated mRNA ac4C modification on *COL5A1* mRNA, RNA decay assay and mRNA translation efficiency assay was performed. The result revealed that overexpression of *NAT10* could maintain *COL5A1* mRNA stability without affecting mRNA translation efficiency (Fig. 1o, p). After getting these results, we detected the expression level of COL1A1, ITGA3, COL6A1 by WB and found a positive correlation of NAT10 with COL6A1 and ITGA3, whereas NAT10 did not regulate the expression of COL1A1 (Supplementary Fig. S11a–d).

COL5A1 was identified as a direct regulatory target of NAT10. However, there are currently no studies that have reported COL5A1 in the study of GC. To clarify this issue, bioinformatics analysis was used and results showed that *COL5A1* expression was significantly elevated in GC tissues (*P* < 0.05), and patients with high *COL5A1* expression were associated with poor OS and PFS (Supplementary Fig. S7a, b). The expression of COL5A1 was significantly positively correlated with the expression of *VIM* and *MMP2*. However, no significant correlation with *CDH1* expression (Supplementary Fig. S7c). qRT-PCR and western blot analysis suggested that *COL5A1* silencing downregulated the expression of VIM and MMP2 (Supplementary Fig. S8a, b). These indicated that COL5A1 may play a promoting role in metastasis and EMT of GC.

In this study, the promoting roles of NAT10 and COL5A1 in the metastasis and EMT of GC were identified. Besides, the regulatory roles of NAT10 in COL5A1 expression were also determined. However, whether the role of NAT10 in promoting GC metastasis and GC cell EMT is attributed to the regulation axis of NAT10/COL5A1 needs to be investigated further. MGC-803 cells and SGC-7901 cells were transfected with lentivirus carrying COL5A1 and/or shNAT10-2 (Supplementary Fig. S9a–d). Cell wound-healing and transwell matrigel invasion assay were performed, and the expression levels of EMT-related proteins measured. The results showed that overexpression of COL5A1 antagonized the suppression of invasion and migration resulted from NAT10 downregulation (Supplementary Fig. S10a–d). Western blot analysis and qPCR assay results indicated that overexpression of COL5A1 also antagonized the suppression of NAT10 downregulation on EMT in GC cells (Supplementary Fig. S9a–d). These results need further verification of the regulatory role of the NAT10/COL5A1 regulation axis in GC metastasis and GC cell EMT. Ulteriorly, we detected the changes of COL5A1 mRNA ac4C modification in MGC-803 and SGC-7901 through acRIP-qPCR. The results showed that the level of COL5A1 mRNA ac4C modification in shNAT10+VEC and shNAT10 + COL5A1 significantly lower than that in shNC+VEC and shNC+COL5A1 (Supplementary Fig. S11 k). However, as for expression level of EMT markers and invasive ability of gastric cancer cells, shNAT10+VEC, shNAT10 + COL5A1, and shNC+shCOL5A1 showed little differences, which indicated that ac4C modification on mRNA participates in maintaining the stability of COL5A1 mRNA whereas exerts no regulatory function of COL5A1 protein activity. Besides, IF assay showed COL5A1, similar to NAT10, could downregulate the expression of CDH2 and VIM, as well as inhibit EMT of gastric cancer. Interestingly, if NAT10 was downregulated, the expression of N-cadherin and VIM with COL5A1 expressed showed no distinct difference compared to the control group (Supplementary Figs. S12 and 13).

In conclusion, this study shows that NAT10 (an mRNA ac4C writing protein) plays a critical role in the process of GC metastasis and GC cell EMT by regulating the mRNA ac4C writing pathway. However, since a large number of genes are involved in the EMT and metastasis of GC cells, the possibility that the NAT10-mediated mRNA ac4C pathway affects the EMT and metastasis of GC cells by regulating other genes cannot be ruled out. This study highlights the importance of ac4C modification on mRNA as a novel gene expression regulation pathway in cancer progression, and provide new insights for further research on the mechanism of tumorigenesis and progression. This study also suggested that targeted inhibition of NAT10 by small-molecule inhibitors may be a potential strategy for the treatment of GC in the future. Due to space limitation, we have added other data, a description of the results of this study, and background introduction and discussion related to this paper to the Supplementary Information.

## Supplementary information

Supplementary materials
