# Supplementary material for: NAT10 promotes gastric cancer metastasis via N4-acetylated COL5A1
Source: Signal Transduct Target Ther. 2021 May 3;6:173. doi: 10.1038/s41392-021-00489-4 (PMC8093205; doi:10.1038/s41392-021-00489-4)
Supplement: Supplementary file 1 — Supplementary materials [file 41392_2021_489_MOESM1_ESM.docx]

Supplementary Materials for

NAT10 promotes gastric cancer metastasis via N4-acetylated COL5A1

**Y-G Zhang^1.2,3#^, Yuanxue Jing^1,3#^, Yinxue Wang^1,3#^, Jianming Tang^4#^, Xiaoran Zhu^1,3^, Wei-Lin Jin^5^, Yiqing Wang^1,3*^, Wenzhen Yuan^1,3*^, Xiangkai Li^6*^ ,Xun Li^1,2,3*^**

^1^The First Hospital of Lanzhou University, 730000, Lanzhou, P.R. China

^2^ Key Laboratory for Biological Therapy and Regenerative Medicine Transformation Gansu Province, 730000, Lanzhou, P.R. China

^3^The First School of Clinical Medicine, Lanzhou University, 730000, Lanzhou, P.R. China

^4^Zhejiang Provincial People's Hospital, 310000, Hangzhou, P.R. China

^5^Institute of Nano Biomedicine and Engineering, Shanghai Engineering Center for Intelligent Diagnosis and Treatment Instrument, Department of Instrument Science and Engineering, Key Laboratory for Thin Film and Microfabrication Technology of Ministry of Education, School of Electronic Information and Electronic Engineering, Shanghai Jiao Tong University, 200240, Shanghai, P. R. China

^6^School of Life Sciences, Lanzhou University,73000, Lanzhou, P.R. China

**This PDF file includes:**

Materials and Methods

Supplementary Text

Figures. S1 to S19

Tables S1 to S4

Data S1.

Materials and Methods

**Patients and specimens**

Our clinical cohort was based on tissue microarrays purchased from Shanghai Outdo Biotech (HStmA 150CS02, Shanghai Outdo Biotech). The microarrays consisted of 75 pairs of GC tissues and matching normal tissues, with complete clinicopathological information (listed in Table S2). The immunohistochemical staining intensity final score method constituted staining intensity scale: 0: no dyeing, 1: weak, 2: middle, 3: strong, the positive score of 0 (positive staining rate < 10), 1 (positive staining rate was 11 to 25), 2 (positive staining rate was 26-50), 3 (positive staining rate was 51-75), and 4 (positive staining rate > 75), the final product of these two was considered as both the expression intensity. The Ethics Committee of the First Hospital of Lanzhou University approved this study.

**Cell culture**

Human gastric epithelial cell line GES-1 and human gastric cancer cell lines SGC-7901, BGC-823, AGS were purchased from Procell Life Science& Technology Co., Ltd. (Wuhan, China). We obtained the MGC-803 cell line from FENGHUISHENGWU (Changsha, China). All the cells were identified through STR and tested for mycoplasma. We routinely cultured all the cells in DMEM medium (L110KJ, Basalmedia) supplemented with 10% fetal bovine serum (FBS) (900-108, GEMINI), and 1% penicillin/streptomycin (S110JV, Basalmedia), incubated in 5% CO_2_, 37℃ incubator (Thermo Scientific, USA) with a humidified and abacterial atmosphere.

**RNA extraction and qRT-PCR**

Total RNA was extracted from the cells using the TRIzon Reagent (CW0580, CWBIO) according to the manufacturer's instructions. gDNA was isolated using the HiFiScript gDNA Removal cDNA Synthesis Kit (CW2582, CWBIO), followed by cDNA synthesis. Expression of RNA was assayed using SYBR Green (CW0760, CWBIO) on the QuantStudio 3 Real-Time PCR Systems (ABI, USA), and the relative RNA expression levels were calculated using the 2^-△△Ct^ method. We utilized theβ-actin gene as an endogenous control. The obtained data were standardized. All the primer sequences are listed in Table S3.

**RIP-qPCR and RIP-seq**

The RIP-qPCR assay was performed using the RIP-kit (Bes5101, BersinBio). Following the manufacturer’s instructions, we carried out the RIP assay in the SGC-7901 and MGC-803 cells to assess and verify the interaction between NAT10 and COL5A1 mRNA. Briefly, magnetic beads pre-coated with 3µg of normal antibodies against mouse immunoglobulin G or NAT10 (ab194297, Abcam) antibodies were incubated with pre-frozen cell lysates (more than 1*10^7^ cells per sample) overnight (>16h) at 4℃. After that, the beads containing immunoprecipitated RNA-protein complexes were subjected to proteinase K digestion, and RNA extraction conducted using the TRIzon Reagent (CW0580, CWBIO). Subsequently, the levels of COL5A1 mRNA were quantitated via RT-qPCR. The total RNA used as input, the endogenous control, and primers for RIP-qPCR are shown in Table S3. Rip-seq was accomplished by Shanghai Genergy Bio.

**acRIP-qPCR and acRIP-seq**

We optimized the Magna meriptmm6A kit (17-10,499, Millipore) protocol by replacing the m6A antibody with the ac4C antibody (ab252215, Abcam). The acRIP analysis of NAT10 knockdown and overexpressed cells was performed. Briefly, the total RNA (200µg) was randomly digested into nucleotide chains of 100-200bp, and a mixture of 5µg ac4C antibody and magnetic beads incubated with the disrupted RNA. Then, the RNAs were purified and analyzed using RT-qPCR. We used the IgG group as the negative control and the input group as the endogenous control. The primers for the acRIP-qPCR are shown in Table S3. acRIP-seq was done by Guangzhou Epibiotek Co., Ltd.

**Luciferase reporter assay**

Following the successful construction of NAT10 silenced and overexpressed cells, for wild-type plasmids, cDNAs containing full-length 3’UTR of COL5A1 were cloned into the pmirGLO vector. For mutant plasmids, the C in the CAC ac4C sequence on the 3’UTR of COL5A1 mRNA was replaced by G. The inserted sequences are listed in Table S4. The wild-type or mutant reporter plasmids and mutant COL5A1 and renilla luciferase reporter vector were co-transfected into SGC-7901-shNC, SGC-7901-shNAT10-2, SGC-7901-Vector, SGC-7901-NAT10, MGC-803-shNC, MGC-803-shNAT10-2, MGC-803-vector, and MGC-803-NAT10, respectively. After 24h, the cells were harvested to evaluate the luciferase enzyme activity on the Varioskan LUX multifunctional enzyme marker apparatus. Moreover, we defined (relative fluorescence intensity)/ (relative RNA expression) as mRNA translation efficiency.

**Construction of stable knockdown and overexpressed cells**

Plasmids expressing shNAT10, shCOL5A1, or shNC, Vector, and NAT10 (OE-NAT10) were synthesized by the PPL company (China). After a large number of plasmids were purified without the endotoxin, NAT10\COL5A1 knockdowns or overexpressed lentiviruses were obtained from the HEK-239T cells using the lentivirus packaging kit (Gmeasy-10, Genomeditech). 1X10^5^ cells were plated into the 6-well plates and transfected with the indicated lentivirus according to the instructions of the manufacturer. Infected cells were selected using 2µg/ml puromycin (ST551-10mg, Beyotime) for 15 days, with the transfection efficiency determined using RT-qPCR and WB analysis. All the targeted sequences are listed in the Table S4.

**Migration and invasion assays**

The effect of NAT10 or COL5A1 on GC cell invasion ability was assessed via the transwell matrigel invasion assay. We added an 8µm aperture Transwell filter (3464, Corning) without a pre-coated diluted Matrigel to 1/8 diluted Matrigel (356234-5m, BD) 100µl for matrigel coating. We placed 5X10^4^ GC cells with Serum-free medium into the upper chamber, and medium containing 10% FBS was added into the bottom chamber, subsequently. After incubating for 24h (NAT10, Vector) or 48h (shNAT10, shNC) at 37℃ with 5% CO_2_, the cells on the underside of the membrane were immobilized and stained with crystal violet (C8470, Solarbio). Then, the penetrated cells were counted in three random fields under a microscope.

**RNA decay assay**

We further evaluated the effect of NAT10 on the stability of COL5A1 mRNA via the RNA decay assay. The SGC-7901-shNC, SGC-7901-shNAT10-2, SGC-7901-Vector, SGC-7901-NAT10, MGC-803-shNC, MGC-803-shNAT10-2, MGC-803-Vector, and MGC-803-NAT10 cells were cultured in 6-well plates. After that, Actinomycin D (M4881, AbMole) was added into each well with a final concentration of 5 µg/ml. The cells were collected after 0, 45, 90, 180, and 360min, respectively. Total RNA was isolated and taken through RT-qPCR to quantify the relative abundance of COL5A1 mRNA. In this test, 18sRNA served as an endogenous control.

**Animal studies**

Four-week-old BALB/c nude mice were used to analyze the effect of NAT10 on in vivo tumorigenesis. Twelve mice were randomly divided into two groups. After that, the SGC-7901-shNAT10-2 cells and SGC-7901-shNC cells (1X10^7^/100µl per mouse, n=6 for each group) were collected and suspended in pre-cooled PBS and subcutaneously injected into mice. The tumor volume changes were closely monitored (volume = length ×width2/2). At the end of the experiment (21 days), the mice were sacrificed, the tumors removed, and used in the HE staining, IHC staining (NAT10, COL5A1, VIM, MMP2, CDH1), WB, and qPCR assays (NAT10, COL5A1, VIM, MMP2, CDH1).

To determine the effect of NAT10 on metastasis, 12 mice were randomly divided into two groups. Each mouse was injected with SGC-7901-shNAT10-2 cells or SGC-7901-shNC cells (5X10^6^/50µl), respectively, through the tail vein. The lungs were removed 21 days later for pulmonary metastatic nodule counting and HE staining.

**Statistical analyses**

Herein, data analyses were conducted using GraphPad Prism 7 and SPSS 19.0 softwares. Differential gene expression analysis and gene expression association analysis were performed using the GEPIA tool. The GC patient survival curves were plotted using the Kaplan-Meier method. A *p*-value of <0.05 was considered to be statistically significant. **p* <0.05, ***p* <0.01, *** *p* <0.001, **** *p* <0.0001, ns means not significant.

Supplementary Text

**Abstract:**

**Background:** Gastric cancer (GC) is among the most prevalent gastrointestinal malignancies. The occurrence of local deep infiltration or distant metastasis in GC is commonly associated with weak treatment and poor prognosis. Although, N4-Acetylcytidine (ac4C) represents one of the extensive chemical modifications in mRNAs that plays a pivotal role in modulating mRNA stability and the mRNA translation process. However, the role of mRNA ac4C modification in disease remains unclear. As the only known ac4C “writer” protein, NAT10 is thought to have critical effects in tumor metastasis and tumor cell epithelial-to-mesenchymal transition (EMT). Here, we report a novel mechanism of NAT10-mediated mRNA ac4C modification regulating gastric cancer metastasis and EMT

**Method:** We performed immunohistochemical (IHC) and bioinformatics analysis to evaluate the correlation between the expression levels of NAT10 and clinical information. *In vitro* and *in vivo* experiments were conducted to investigate the effect of NAT10 on GC metastasis. RIP-seq, acRIP-seq, a luciferase reporter assay, RNA stability assay, RNA translation efficiency assay, and other techniques were utilized to elucidate the regulatory mechanism of NAT10 in GC metastasis.

**Result:** NAT10 was significantly up-regulated in GC. Clinically, NAT10 up-regulation was associated with poor prognosis and lymph node metastasis. The cell and animal experiment findings showed that NAT10 enhanced the EMT and metastasis of cancer cells *in vivo*. Mechanistically, we established that NAT10 up-regulated the modification levels of COL5A1 mRNA ac4C, maintained the stability of COL5A1, and up-regulated the expression of COL5A1, further promoting GC cell EMT, which led to GC metastasis.

**Conclusion:** Herein, we propose a novel mechanism through which mRNA ac4C modification regulates cancer metastasis. Besides, we demonstrate the regulation effect of NAT10-COL5A1 on GC cell EMT and GC metastasis. Therefore, we provide a potential therapeutic strategy for GC.

**Key Words:**

Gastric cancer; RNA modification; N4-acetyclytidine (ac4C); metastasis; Epithelial-mesenchymal transformation (EMT)

.

**Introduction**

Human gastric cancer is the sixth most common malignancy，and about 1,033,701 new cases and 782,685 deaths were reported in 2018 [1] .GC is the second leading cause of cancer-related deaths globally [1]. Surgery remains the only promising curative treatment for GC, however, metastasis is the bottleneck in GC treatment [2]. Therefore, understanding the molecular mechanisms underlying metastasis in GC may reveal new therapeutic targets for metastatic GC.

Epithelial-mesenchymal transition (EMT) reduces the intercellular adhesion of epithelial cells and up-regulate cell movement and invasion [3]. During the process of cancer initiation and progression, EMT promotes tumor cells' drug resistance [4]. EMT is considered to be a direct cause of tumor metastasis [5, 6], it is also known to cause remodeling of ECM (collagen, tenascin, etc.) [7] and promotion of tumor metastasis [8]. The transformation of ECM can also change EMT-related signal transduction and protein expression in tumor cells by interacting with receptors on the cell membrane and other cell components, to promote EMT [9]. The COL5A1 gene encodes the α1 chain for one of the low abundance fibrillar collagen.

In this study, TCGA datasets were queried to explore the expression level of COL5A1 in 23 common tumors. The results revealed that the expression levels of COL5A1 were significantly up-regulated in 12 common tumors, including BRCA, CHOL, COAD, ESCA, HNSC, KIRC, LUAD, LUSC, READ, SARC, STAD, UCEC. (Fig. S14,15). The researches before showed that COL5A1 signs significant responsible in tumor metastasis [10] and hypertrophic scar formation of myocardial cells [11]. Besides, there is study has found COL5A1 a marker of EMTⅡ in tumor cells for it could promotes EMT directly [12, 13]. Consistently, Liu W. et al. [14] showed that COL5A1 contributed to the metastasis of lung adenocarcinoma. The high expression of COL5A1 has been associated with poor clinical prognosis in breast cancer [15], kidney cancer [16], and tongue squamous cell carcinoma [17]. In this study, the expression of COL5A1 mRNA was found to be mainly elevated in GC samples, which predicted poor overall survival. *In vitro* experiments showed that COL5A1 up-regulated the expression levels of key EMT proteins, MMP2 and VIM, and promoted the migration and invasion of GC cells. However, the imbalance in COL5A1 expression in GC remains unclear.

Current studies show that mRNA modifications play a pivotal role in mRNA splicing, stability, subcellular localization, and translation [18-21]. There are numerous complex chemical modifications on mRNA, including m6A [22], m6Am [23], m1A [24], m5C [25] and ac4C [26], etc. However, numerous studies have mainly focused on m6A modification in various diseases. Cancer initiation and progression are often accompanied by general mRNA modification or key proteins of mRNA modification expression disorders. Li et al. [27] reported that FTO plays an oncogenic role in acute myeloid leukemia as an m6A demethylase and in dysregulation of the expression of m6A Methyltransferase METTL3 which promotes the progression of liver cancer and GC [28, 29]. m6A reading protein YTHDF1 promotes the progression of ovarian cancer through translational regulation [30]. The dysregulation of m5C modification was also reported to play an important role in the progression of bladder cancer [26] The main known mRNA modifications, their corresponding modification proteins, biological functions, and research in cancer are summarized in table S1.

In 2018, the U.S. National Cancer Institute (NCI)[26], for the first time identified the N4-acetylcytidine (ac4C) and considered NAT10 as the “writer” protein of acetylation on mRNA. Functionally, ac4C modification is believed to maintain RNA stability and promote RNA translation in mRNA metabolism, and upregulate the expression of the target gene. Very recently, acetylation of cytidine (ac4C) was identified as a new mRNA modification that increases the stability and translation efficiency of transcripts. N-acetyltransferase 10 (NAT10) is the only known mRNA ac4C “writer” protein, that has been widely studied. NAT10 is reported to be involved in aging [51], tumors [52], and in multiple biological processes. NAT10 also promotes tumor cell EMT, leading to tumor metastasis and resistance to chemotherapy [53, 54]. However, there are no studies that have discussed the role and mechanism of NAT10-mediated mRNA acetylation modification (ac4C modification) in the above stduies. Using the TGCA database, the expression of NAT10 in 23 common tumors (Fig. S16,17) was analyzed. The expression of NAT10 was significantly up-regulated in STAD, CHOL, READ, and COAD. Further analysis of the KM-plotter database revealed that high NAT10 expression in GC was associated with poor prognosis of GC patients. Moreover, *in vitro* studies show that NAT10, like COL5A1, can promote the expression of MMP2 and VIM in GC cells, and give rise to GC metastasis. *In vivo* studies reveal that knockdown of NAT10 inhibits the metastasis of GC and down-regulates the expression of VIM and MMP2. Molecular experiments have revealed that COL5A1 is the direct target of NAT10, and NAT10 maintains the stability of COL5A1 mRNA through the ac4C modification dependent pathway and up-regulates the expression level of COL5A1 in GC cells.

**Results**

**NAT10 is Highly Expressed in GC**

The analysis of RNA-seq data in the TCGA STAD dataset revealed that the expression of *NAT10* in GC was significantly up-regulated compared with normal tissues (P<0.05) (Fig. S1a). Next, the correlation between *NAT10* expression and clinical prognosis of GC patients was analyzed using Kaplan-Meier survival analysis. The results suggested that GC patients with high *NAT10* expression exhibited a significantly poorer OS (overall survival), PPS (post-progression survival), and FP (first progression) than those with low *NAT10* expression (Fig. S1b, c, d). Immunohistochemical (IHC) analysis was performed to assess the NAT10 expression in 75 GC tissues and their corresponding para-cancer tissues (Fig. S1e, f), and to determine the correlation between the expression level of NAT10 in cancer tissues and clinical data at the protein level. The expression of NAT10 in GC tissues was found to be upregulated, and the expression level of NAT10 was correlated with the AJCC stage and lymph node metastasis (Table. S2). Western blot analysis and qPCR assays were performed to detect NAT10 expression level in gastric epithelial cells GES-1 and 4 GC cell lines (SGC-7901， BGC-823， MGC-803 and AGS). The results showed that NAT10 expression level was upregulated at the protein level in GC cell lines compared with the gastric epithelial cell lines (Fig. S1g). However, at the RNA level, *NAT10* was only expressed at a significantly higher level in SGC-7901 and MGC-803 cell lines compared with GES-1 cell lines (Fig. S1h). Therefore, subsequent experiments were conducted based on SGC-7901 and MGC-803 cells. Results showed that NAT10 is frequently up-regulated in human GC and may be implicated in staging and progression of GC. Base on the results above, we utilized cell wound healing and transwell matrigel invasion assays to evaluate the invasion and migration ability of MGC-803, BGC823, SGC7901 and AGS cells. The result showed that as for invasion and migration, AGS is better than MGC-803, BGC-823 and SGC-7901. However, considering the cell culture medium of AGS is F12, which is different from the other cells, we believe that AGS is not comparable (Fig. S11e-h).

**NAT10 Enhances GC Cell Migration and Invasion *in Vitro***

Cancer metastasis poses major challenges in the clinical treatment of GC. To investigate the role and mechanism of *NAT10* in GC metastasis, sh-*NAT10-*1, sh-*NAT10*-2, sh-NC, Vector, and *NAT10*-overexpressing plasmid vectors, were established. Lentivirus infection technology was used to generate corresponding stable cell lines. The modified NAT10 expressions were confirmed by both western blot analysis and qPCR. The results showed that the silencing effect of sh*NAT10*-2 plasmid on *NAT10* was significantly higher than that of sh*NAT10*-1 (Fig. S2a, b). Based on these results, the sh*NAT10*-2 plasmid was selected for subsequent experiments. In the *in vitro* GC model, wound healing assay demonstrated that the silencing of *NAT10* significantly impeded the migratory ability of SGC-7901 and MGC-803 cells, while overexpression of *NAT10* enhanced the migratory ability of SGC-7901 and MGC-803 cells (Fig. S2c, d). Moreover, transwell assay showed that knockdown of *NAT10* highly suppressed GC cell invasive abilities, while it was markedly promoted by overexpression of *NAT10* (Fig. S2e). Taken together, these results indicate that NAT10 plays a promoting role *in vitro* migration and invasion of GC cells.

**NAT10 Contributes to GC Progression *in vivo***

To evaluate the regulatory role of NAT10 in GC, a subcutaneous implantation experiment in nude mice was performed. Besides, tail vein injection increased lung colonization to explore the regulatory effects of NAT10 on tumor formation, growth, and metastasis in GC. The results of the subcutaneous implantation experiment showed that the tumor volume and mass of the experimental group injected with stable SGC-7901-sh*NAT10*-2 cells were significantly lower compared with the control group injected with SGC-7901-shNC cells (Fig. S3a). The tail vein pulmonary metastasis test showed that the down-regulation of *NAT10* expression in SGC-7901 cells inhibited lung metastasis of SGC-7901 cells. The number of pulmonary metastatic nodules was significantly lower in the experimental group injected with SGC-7901-sh*NAT10*-2 compared with the control group (Fig. S3d, e). The above results indicated that NAT10 played a promoting role in tumor formation, growth, and metastasis of GC cells *in vivo*. IHC, WB, and qPCR were used to detect the expression level of NAT10 in the tumor tissues obtained from the subcutaneous tumor formation experiment (Fig. S3b, c).

**NAT10 Promotes EMT in GC Cells**

EMT is the direct cause of tumor metastasis. To explore the relationship between NAT10 and EMT, the effect of NAT10 expression on EMT marker gene expression was first determined. The expression of EMT marker protein CDH1, MMP2, VIM was determined by both qRT-PCR and western blot analysis, in SGC-7901 and MGC-803 cell lines of *NAT10* overexpression or knockdown. The result showed that the silencing of *NAT10* decreased the expression levels of VIM and MMP2 at both the protein and RNA levels, but CDH1 expression was not affected. Accordingly, the overexpression of *NAT10* up-regulated the expression levels of VIM and MMP2 except for CDH1 (Fig. S4a, b, c, d). IHC, WB, and qPCR assay were performed *in vivo*, using tumor tissues obtained from the subcutaneous tumorigenesis experiment. The results indicated that VIM and MMP2 expressions were down-regulated in the experimental group injected with *NAT10* knockdown SGC-7901 cells. Similarly, there was no significant change in CDH1 expression (Fig. S4e, f, g). VIM and MMP2 are known to play a promoting role in the regulation of EMT, while CDH1 has an inhibitory effect. These results demonstrated that *NAT10* was positively correlated with the expression of VIM and MMP2, but CDH1 exhibited no obvious relationship. Therefore, NAT10 was suggested to play a promoting role in the EMT process of GC cells. Simultaneously, IF was performed to clarify the effect of changing the NAT10 expression on CDH1, CDH2 and VIM. The result showed that NAT10 is positively related to CDH2 and VIM, while the expression of CDH1 has little change, which indicate that NAT10 could promote EMT of gastric cancer cells (Fig. S12,13)

**COL5A1 Is a Direct Regulatory Target of NAT10**

To explore the regulatory role of NAT10 in EMT and metastasis of GC, and identify its potential targets, acRIP-seq was used to compare the ac4C modified profile following *NAT10* knockdown in SGC-7901 cells. Besides, the KEGG pathway enrichment analysis was performed (Fig. S5a, b). The ac4C modification down-regulated genes were significantly enriched in gene sets involved in the ECM pathway and the cell adhesion pathway after *NAT10* silencing (Fig. S5c). RIP-seq was performed to investigate the RNA molecules interacting with NAT10 in SGC-7901 cells. The results revealed that there were 10709 RNAs bound and interacted with NAT10 in SGC-7901 cells (Table S5), suggesting that NAT10 is widely involved RNA-binding protein, and may play a pivotal role in the regulation of cell life activities through RNA binding. Comprehensive analysis of acRIP-seq and RIP-seq data showed that NAT10 was significantly bound and interacted with *COL5A1* mRNA, and *NAT10* silencing down-regulated the ac4C modification on *COL5A1* mRNA 3’UTR region (Fig. S5d, e). More importantly, the gene function of *COL5A1* was enriched in gene sets involved in the ECM and cell adhesion pathways, indicating that *COL5A1* may play a significant role in the metastasis of GC. Next, we used acRIP-qPCR and RIP-qPCR to verify the interaction between NAT10 and *COL5A1* mRNA, and also identify the regulatory effect of NAT10 on ac4C modification of *COL5A1* mRNA. The RIP-qPCR assay revealed that NAT10 in both SGC-7901 cells and MGC-803 cells showed strong binding and interaction with *COL5A1* mRNA. The acRIP-qPCR assay showed the decreasing of the abundance of ac4C modification on COL5A1 mRNA in NAT10-knocked down SGC-7901 and MGC-803 cells. Besides, the up-regulated expression of *NAT10* up-regulated the abundance of ac4C modification on *COL5A1* mRNA (Fig. S5f, g). In conclusion, *COL5A1* was identified as a direct target of NAT10-mediated mRNA ac4C modification. On the basis of the experiments above, conducting homology analysis didn’t find the homology of COL5A1 with ITGA3, COL6A1, COL1A1 (Fig. S11i, j).

***NAT10* promotes *COL5A1* expression**

NAT10 is known to regulate ac4C modification on *COL5A1* mRNA 3’UTR through direct interaction, however, the biological function of this regulation remains unknown. To understand the regulatory role of NAT10 on *COL5A1*, qPCR and western blot analysis were conducted *in vitro* GC models. Knockdown of *NAT10* significantly reduced COL5A1 expression at both the RNA and protein levels, and overexpression of *NAT10* up-regulated the expression of COL5A1 (Fig. S6a, b). Next, the luciferase reporter gene assay was used to investigate whether *NAT10* regulates the expression level of *COL5A1* through the ac4C modification pathway of mRNA. Both wild-type and mutant *COL5A1* reporter genes were constructed. For the mutant form of *COL5A1*, the ac4C consensus sequences CAC were replaced by GAG, thus abolishing ac4C modification (Fig. S6d). Luciferase activity of the wild-type *COL5A1* reporter was also significantly augmented upon *NAT10* overexpression, and the silencing of *NAT10* down-regulated the relative fluorescence intensity. Knockdown or overexpression of *NAT10* showed no significant effect on the expression of the mutant *COL5A1* reporter (Fig. S6e). Luciferase reporter gene assay further exhibited that *NAT10* had a positive regulatory effect on *COL5A1* expression, and this regulatory effect was completed by ac4C modification on *COL5A1* 3'UTR. To further assess the effect of *NAT10*-mediated mRNA ac4C modification on *COL5A1* mRNA, RNA decay assay, and mRNA translation efficiency assay (We defined relative fluorescence intensity/relative RNA expression as mRNA translation efficiency) were performed. The result revealed that overexpression of *NAT10* could maintain *COL5A1* mRNA stability without affecting mRNA translation efficiency (Fig. S6c, f). In the *in vivo* GC model, IHC, WB, qPCR experiments were performed using tumor tissues from subcutaneous tumorigenesis experiments. The expression level of COL5A1 in the experimental group injected with SGC-7901-sh-*NAT10*-2 cells and the control group injected with SGC-7901-sh-NC cells was validated. The results showed that knocking down *NAT10* inhibited the expression of COL5A1 (Fig. S6g, h, i). Therefore, it was suggested that NAT10 promoted the expression of COL5A1 both *in vivo* and *in vitro* GC models, which is dependent on the promotion of *COL5A1* mRNA stability by ac4C modification on *COL5A1* mRNA 3'UTR. However, ac4C modification on *COL5A1* 3'UTR did not regulate the translation efficiency of *COL5A1* mRNA. After getting these results, we detected the expression level of COL1A1, ITGA3, COL6A1 by WB and qRT-PCR, and found positive correlation of NAT10 with COL6A1 and ITGA3, whereas NAT10 didn’t regulate the expression of COL1A1 (Fig. S11a-d).

**COL5A1 Plays a Promoting Role in GC Progression**

Current studies suggest that remodeling of extracellular ECM has a pivotal effect on tumor metastasis. However, there are currently no studies that have reported COL5A1 in the study of GC. Therefore, in this study, the RNA-seq data in TCGA STAD dataset was analyzed and results showed that *COL5A1* expression was significantly elevated in GC tissues (P<0.05) (Fig. S7a). KM-PLOT database revealed that patients with high *COL5A1* expression were associated with poor OS and PFS (Fig. S7b). Meanwhile, gene association analysis of the RNA-seq data set related to GC tissues in the TCGA database was performed and the results indicated that the expression of COL5A1 was significantly positively correlated with the expression of *VIM* and *MMP2*. However, no significant correlation with *CDH1* expression was reported, which is similar to the effect of *NAT10* expression on *CDH1\MMP2\VIM* expression (Fig. S7c). This indicates that COL5A1 may play a promoting role in metastasis and EMT of GC. A stable cell line with down-regulated *COL5A1* expression in SGC-7901 and MGC-803 cells was constructed, and cell wound healing and transwell matrigel invasion assays were performed. Wound healing assay showed that down-regulation of *COL5A1* significantly inhibited the migration of GC cells (Fig. S8c), while transwell matrigel invasion assay revealed that down-regulation of *COL5A1* expression inhibited the invasion ability of GC cells (Fig. S8d). qRT-PCR and western blot analysis was performed, to determine the effect of *COL5A1* silencing on the expression of EMT markers VIM, MMP2, and CDH1. The results suggested that *COL5A1* silencing down-regulated the expression of VIM and MMP2, but did not affect CDH1 expression (Fig. S8a, b). Therefore, COL5A1 was suggested to play a similar promoting role to NAT10 in the metastasis of GC.

**COL5A1 Is an Intermediate Medium of NAT10 Regulates GC Metastasis and GC Cell EMT**

In this study, the promoting roles of NAT10 and COL5A1 in the metastasis and EMT of GC were identified. Besides, the regulatory roles of NAT10 in COL5A1 expression was also determined. However, whether the role of NAT10 in promoting GC metastasis and GC cell EMT is attributed to the regulation axis of *NAT10/COL5A1* needs to be investigated further. MGC-803 cells and SGC-7901 cells were transfected with lentivirus carrying *COL5A1* and/or sh-*NAT10*-2 (Fig. S9 a-d). Cell wound healing and transwell matrigel invasion assay were performed, and the expression levels of EMT - related proteins measured. The results showed that overexpression of *COL5A1* antagonized the suppression of invasion and migration resulted from *NAT10* downregulation (Fig. S10 a-d). Western blot analysis and qPCR assay results indicated that overexpression of COL5A1 also antagonized the suppression of *NAT10* down-regulation on EMT in GC cells (Fig. S9 a-d). These results need further verification of the regulatory role of the *NAT10/COL5A1* regulation axis in GC metastasis and GC cell EMT. Ulteriorly, we detected the changes of COL5A1 mRNA ac4C modification in MGC-803 and SGC-7901 through acRIP-qPCR. The results showed that the level of COL5A1 mRNA ac4C modification in shNAT10+VEC and shNAT10+COL5A1 significantly lower than that in shNC+VEC and shNC+COL5A1(Fig. S11k). However, as for expression level of EMT markers and invasive ability of gastric cancer cells, shNAT10+VEC, shNAT10+COL5A1 and shNC+shCOL5A1 showed little differences, which indicated that ac4C modification on mRNA participates in maintaining the stability of COL5A1 mRNA whereas exerts no regulatory function of COL5A1 protein activity. Besides, IF assay showed COL5A1, similar to NAT10, could downregulate the expression of CDH2 and VIM, as well as inhibit EMT of gastric cancer. Interestingly, if NAT10 was downregulated, the expression of CDH2 and VIM with COL5A1 expressed showed no distinct difference compared to the control group (Fig. S12,13).

**Discussion**

mRNA modification is one of the most common chemical modifications in mRNA. With the recent advances in biochemistry and high-throughput sequencing technology, researchers can precisely quantify and locate the amount of mRNA modification, determine the modification sites, and reveal the functions in biological regulation and disease progression [26, 29, 55]. Increasing evidence suggests that mRNA modification is widely dysregulated during tumor tumorigenesis and development. However, mRNA modification-related research has mainly focused on m6A, and other modifications (such as m1A、m5C、m6Am、ac4C, etc.) have rarely been studied for their potential effects and mechanisms in other diseases [31, 56, 57]. In this study, the expression of NAT10, the only known mRNA ac4C “writer” protein, was found to increase significantly in GC tissues compared with normal gastric tissues. Particularly, this up-regulation was associated with poor survival prognosis, lymph node metastasis, and cancer stage in GC patients. Together, these findings indicate that NAT10 is an unfavorable factor affecting GC patient prognosis, may serve as a biomarker for tumorigenesis and progression of GC, or can be a potential target for diagnosis and pharmaceutical drug development against GC in the future.

Stable cell lines with the expression of NAT10 changes were successfully constructed, to verify whether NAT10 participated in the regulation of GC metastasis, and the effects of NAT10 expression on the migration and invasion of GC cells were also detected. The results showed that NAT10 promoted invasion and migration in GC cells. The effect of NAT10 expression on EMT in GC cells was also tested. The results confirmed that NAT10 was positively correlated with EMT in GC cells. Similarly, NAT10 has been reported to be positively correlated with EMT in liver cancer and breast cancer [53, 58]. *In vivo* studies used the subcutaneous tumorigenesis experiments to detect the variation in subcutaneous tumor formation ability of GC cells after the downregulation of NAT10, and the tail vein injection to analyze lung colonization, and detect the metastasis ability of GC cells after downregulation of NAT10. The results indicated that depleted expression of NAT10 significantly inhibited the tumorigenicity and metastasis of GC cells in mice. Moreover, IHC detection also indicated that NAT10 promoted the EMT of GC cells *in vivo*. Based on these results, NAT10 was found to promote metastasis of GC through EMT at the cellular level. However, to determine, the regulation mode and NAT10 pathway in GC metastasis need further investigations.

Previous studies have shown that NAT10 promotes tumor metastasis and drug resistance through EMT [53, 58], however, the molecular mechanism remains unknown. Besides, whether mRNA ac4C modification is involved in the regulation of these processes remains unclear. Previous studies suggest that ac4C (N-4 acetylcytidine) in body fluids is a potential disease marker [59, 60]. In cancer, autoimmune diseases, and metabolic diseases, the relative abundance of ac4C can be used to predict the occurrence and development of the disease to a certain extent [61-63]. However, previous studies in molecular biology showed that ac4C modification is mainly located in non-coding RNAs such as tRNA and rRNA [64-66]. In 2018, Arango et al. reported for the first time, the existence of acetylation modification (N-4-acetylcytirin, ac4C) on mRNA, which can promote RNA translation and maintain RNA stability [26]. However, it is unknown whether mRNA ac4C modification plays a regulatory role in human diseases, especially in tumor tumorigenesis and development. To further reveal the related biological action mechanism, RIP-seq and acRIP-seq were performed, and comprehensive analysis identified COL5A1 as an intermediate molecular target of NAT10 to promote GC metastasis. The analysis revealed that NAT10 was extensively bound to and interacted with RNA molecules, indicating that NAT10 may play a key regulatory role in cell life activities through the action mode of RNA binding proteins. acRIP-seq combined with KEGG pathway enrichment analysis indicated that the down-regulated ac4C modified genes were mainly concentrated in the ECM pathway and the adhesion spot pathway after the downregulation of NAT10 (the KEGG pathway enrichment of COL5A1 was also concentrated in both the ECM pathway and the adhesion spot pathway). The ECM and the adhesion spot pathways played a pivotal role in the process of cancer cell metastasis and EMT [7], which indicated that NAT10 may have a significant effect on GC metastasis through mRNA ac4C.

Our studies have revealed that NAT10 can regulate the modification of *COL5A1* mRNA ac4C through direct interaction. However, the biological effects of this regulation remain unknown. qPCR, WB, and IHC were used to detect the COL5A1 expression with the change of NAT10 expression. The results showed that the expression of NAT10 was positively correlated with the expression of COL5A1. The luciferase report assay also indicated that NAT10 directly regulated the expression of COL5A1 and that this was dependent on the modification of ac4C on *COL5A1* mRNA 3’UTR. RNA stability experiments showed that the expression of NAT10 was positively correlated with the stability of *COL5A1* mRNA. Based on these experimental results, NAT10 was suggested to regulate the stability of *COL5A1* mRNA rather than the translation efficiency by regulating the modification of ac4C on *COL5A1* mRNA, thus up-regulating the expression level of COL5A1. These findings are consistent with previous research results reported by Arango et al. that ac4C modification on mRNA can up-regulate the expression level of target genes [26].

mRNA ac4C modification is the core of this study. Arango et al. suggested that ac4C on mRNA was bound to the CDS region of the mRNA [26]. However, this study showed that ac4C on *COL5A1* mRNA was bound in the 3’UTR region of the mRNA. Moreover, these results have shown that ac4C modification located in the 3’UTR region on *COL5A1* mRNA can only regulate the stability of *COL5A1* mRNA, but not the efficiency of RNA translation. The 4 NAT10 potential regulatory targets obtained by combining RIP and acRIP screening in cancer metastasis had ac4C modification at the 3’UTR region on the mRNA (Figure. S18,19). In this study, it was hypothesized that the modification of ac4C in different mRNA segments may play different regulatory roles in mRNA fate determination. Specifically, ac4C modification located in the CDS region near the initial codon may affect the mRNA translation regulation process, however, the ac4C modification at 3’UTR plays a regulatory role in mRNA stability. This study was only limited to the regulatory relationship between NAT10 and COL5A1 and did not verify this phenomenon on a larger scale, nor discuss the underlying molecular mechanism. Therefore, future studies should further explore the regulatory function and mechanism of ac4C modification from a molecular biology perspective. Besides, part of the mRNA in SGC-7901 cells has ac4C modification after NAT10 down-regulation. More surprisingly, the degree of ac4C modification enrichment was up-regulated on some mRNAs after the downregulation of NAT10. Arango et al. also showed that about 20% of the mRNA ac4C modification remained in the cells after NAT10 knockout, suggesting that there are other mRNA ac4C “writer” proteins in human cells besides NAT10 [26]. Similar to the modification of m6A [41, 32], m6Am [44], and m1A [48, 67], there may exist an “eraser” of ac4C on mRNA. Therefore, ac4C on mRNA may be a dynamic process regulated by the combination of the “writer” proteins and the “eraser” proteins. Additionally, similar to m6A modification [19, 21, 36-39, 68], m5C modification [31, 50] and m1A modification [47], ac4C modification on mRNA was speculated to rely on the “reader” protein to regulate the fate of RNA. This study revealed that NAT10 maintained the stability of COL5A1 mRNA and promoted its expression through ac4C modification in GC cells. However, the recognition proteins that interacted with the ac4C-modified COL5A1 mRNA to maintain its stability remain unstudied. In this study, RNA-binding proteins that maintain the stability of COL5A1 mRNA by RNA-pulldown were explored. However, due to the special chemical structure modified by ac4C, the modification of ac4C on mRNA was extremely unstable *in vitro*, and a stable RNA probe with ac4C modification could not be obtained, and this led to the failure of subsequent experiments. Further investigation to reveal the regulation mechanism and function of ac4C modification depends on more advanced RNA synthesis and RNA-protein interaction analysis methods and to also explore the recognition proteins modified by ac4C.

COL5A1 is a component of rare extracellular collagen. This study identified COL5A1 as the downstream target of NAT10-mediated mRNA ac4C modification regulating GC metastasis. However, the regulatory role of COL5A1 was initially unclear. KEGG pathway enrichment analysis revealed that COL5A1 plays an important role in ECM and adhesion plaque pathways. ECM changes play a critical role in tumor angiogenesis and tumor metastasis [69]. This study showed that COL5A1 was positively correlated with the expression of MMP2 and VIM, and promoted GC cell EMT and metastasis. COL5A1 also plays an upstream regulatory role in the process of EMT. It has been reported that ECM regulates intracellular signal transduction by binding and interacting with cell receptors, thus inducing EMT and leading to cancer metastasis [70]. Currently, studies show that ECM component change is an important regulatory node for cell migration and invasion [71, 72]. Besides, the changes of the ECM matrix also promotes the formation of intestinal embryos and the migration of neurospinocytes in normal physiological processes, and are involved in complex EMT regulation [73, 74]. Unfortunately, this study is only based on cell level experiments and bioinformatics prediction, focusing on the regulation of the COL5A1 molecular process by mRNA ac4C modification of NAT10. The study reveals that COL5A1 plays an important role in the process of GC cell EMT, and COL5A1 is located in the downstream of the regulation axis of NAT10-COL5A1. However, the study does not explore the specific mechanisms of COL5A1 regulation of GASTRIC cancer EMT.

In conclusion, this study shows that NAT10 (an mRNA ac4C writing protein) plays a critical role in the process of GC metastasis and GC cell EMT by regulating the mRNA ac4C writing pathway. However, since a large number of genes are involved in the EMT and metastasis of GC cells, the possibility that NAT10-mediated mRNA ac4C pathway affects the EMT and metastasis of GC cells by regulating other genes cannot be ruled out. This study highlights the importance of ac4C modification on mRNA as a novel gene expression regulation pathway in cancer progression, and provide new insights for further research on the mechanism of tumorigenesis and progression. This study also suggested that targeted inhibition of NAT10 by small-molecule inhibitors may be a potential strategy for the treatment of GC in the future.

**Abbreviations**

NAT10 N-acetyltransferase 10

ac4C N4-acetylcytidine

COL5A1 Collagen Type V Alpha 1 Chain

GC gastric cancer

STAD Stomach adenocarcinoma

COAD Colon adenocarcinoma

CHOL Cholangio carcinoma

READ Rectum adenocarcinoma

EMT Epithelial-Mesenchymal Transition

RIP RNA Immunoprecipitation

acRIP ac4C-RNA Immunoprecipitation

TCGA The Cancer Genome Atlas

GEPIA gene expression profiling and interactive analyses

shRNA short hairpin RNA

qRT-PCR Quantitative real-time PCR

WB Western blot

IHC Immunohistochemistry

3’UTR 3’untranslated region

**References**

1. Patasius, A. & Smailyte, G. Re: MaryBeth B. Culp, Isabelle Soerjomataram, Jason A. Efstathiou, Freddie Bray, Ahmedin Jemal. Recent Global Patterns in Prostate Cancer Incidence and Mortality Rates. Eur Urol 2020;77:38-52. Eur. Urol. 77, e132 (2020).

2. Kim, Y. M. & Hyung, W. J. Current status of robotic gastrectomy for gastric cancer: comparison with laparoscopic gastrectomy. Updates Surg , (2021).

3. Lamouille, S., Xu, J. & Derynck, R. Molecular mechanisms of epithelial-mesenchymal transition. Nat. Rev. Mol. Cell Biol. 15, 178-196 (2014).

4. Arumugam, T. et al. Epithelial to mesenchymal transition contributes to drug resistance in pancreatic cancer. Cancer Res. 69, 5820-5828 (2009).

5. Tsai, J. H., Donaher, J. L., Murphy, D. A., Chau, S. & Yang, J. Spatiotemporal regulation of epithelial-mesenchymal transition is essential for squamous cell carcinoma metastasis. Cancer Cell 22, 725-736 (2012).

6. Thiery, J. P. Epithelial-mesenchymal transitions in tumour progression. Nat. Rev. Cancer 2, 442-454 (2002).

7. Peixoto, P. et al. EMT is associated with an epigenetic signature of ECM remodeling genes. Cell Death Dis 10, 205 (2019).

8. Yang, J. et al. Twist, a master regulator of morphogenesis, plays an essential role in tumor metastasis. Cell 117, 927-939 (2004).

9. Hoshiba, T. An extracellular matrix (ECM) model at high malignant colorectal tumor increases chondroitin sulfate chains to promote epithelial-mesenchymal transition and chemoresistance acquisition. Exp. Cell Res. 370, 571-578 (2018).

10. Liu, W. et al. COL5A1 may contribute the metastasis of lung adenocarcinoma. Gene 665, 57-66 (2018).

11. Yokota, T. et al. Type V Collagen in Scar Tissue Regulates the Size of Scar after Heart Injury. Cell 182, 545-562.e23 (2020).

12. Vittal, R. et al. IL-17 induces type V collagen overexpression and EMT via TGF-β-dependent pathways in obliterative bronchiolitis. Am. J. Physiol. Lung Cell Mol. Physiol. 304, L401-414 (2013).

13. Kinker, G. S. et al. Pan-cancer single-cell RNA-seq identifies recurring programs of cellular heterogeneity. Nat. Genet. 52, 1208-1218 (2020).

14. Liu, W. et al. COL5A1 may contribute the metastasis of lung adenocarcinoma. Gene 665, 57-66 (2018).

15. Wu, M. et al. Prospective molecular mechanism of COL5A1 in breast cancer based on a microarray, RNA sequencing and immunohistochemistry. Oncol. Rep. 42, 151-175 (2019).

16. Feng, G. et al. Overexpression of COL5A1 promotes tumor progression and metastasis and correlates with poor survival of patients with clear cell renal cell carcinoma. Cancer Manag Res 11, 1263-1274 (2019).

17. Chen, H. C. et al. Differential clinical significance of COL5A1 and COL5A2 in tongue squamous cell carcinoma. J. Oral Pathol. Med. 48, 468-476 (2019).

18. Molinie, B. et al. m(6)A-LAIC-seq reveals the census and complexity of the m(6)A epitranscriptome. Nat. Methods 13, 692-698 (2016).

19. Wang, X. et al. N6-methyladenosine-dependent regulation of messenger RNA stability. Nature 505, 117-120 (2014).

20. Zheng, G. et al. ALKBH5 is a mammalian RNA demethylase that impacts RNA metabolism and mouse fertility. Mol. Cell 49, 18-29 (2013).

21. Wang, X. et al. N(6)-methyladenosine Modulates Messenger RNA Translation Efficiency. Cell 161, 1388-1399 (2015).

22. Dominissini, D. et al. Topology of the human and mouse m6A RNA methylomes revealed by m6A-seq. Nature 485, 201-206 (2012).

23. Mauer, J. et al. Reversible methylation of m6Am in the 5' cap controls mRNA stability. Nature 541, 371-375 (2017).

24. Dominissini, D. et al. The dynamic N(1)-methyladenosine methylome in eukaryotic messenger RNA. Nature 530, 441-446 (2016).

25. Squires, J. E. et al. Widespread occurrence of 5-methylcytosine in human coding and non-coding RNA. Nucleic Acids Res. 40, 5023-5033 (2012).

26. Arango, D. et al. Acetylation of Cytidine in mRNA Promotes Translation Efficiency. Cell 175, 1872-1886.e24 (2018).

27. Li, Z. et al. FTO Plays an Oncogenic Role in Acute Myeloid Leukemia as a N6-Methyladenosine RNA Demethylase. Cancer Cell 31, 127-141 (2017).

28. Chen, M. et al. RNA N6-methyladenosine methyltransferase-like 3 promotes liver cancer progression through YTHDF2-dependent posttranscriptional silencing of SOCS2. Hepatology 67, 2254-2270 (2018).

29. Yue, B. et al. METTL3-mediated N6-methyladenosine modification is critical for epithelial-mesenchymal transition and metastasis of gastric cancer. Mol. Cancer 18, 142 (2019).

30. Liu, T. et al. The m6A reader YTHDF1 promotes ovarian cancer progression via augmenting EIF3C translation. Nucleic Acids Res. 48, 3816-3831 (2020).

31. Chen, X. et al. 5-methylcytosine promotes pathogenesis of bladder cancer through stabilizing mRNAs. Nat. Cell Biol. 21, 978-990 (2019).

32. Bokar, J. A., Shambaugh, M. E., Polayes, D., Matera, A. G. & Rottman, F. M. Purification and cDNA cloning of the AdoMet-binding subunit of the human mRNA (N6-adenosine)-methyltransferase. RNA 3, 1233-1247 (1997).

33. Liu, J. et al. A METTL3-METTL14 complex mediates mammalian nuclear RNA N6-adenosine methylation. Nat. Chem. Biol. 10, 93-95 (2014).

34. Schwartz, S. et al. Perturbation of m6A writers reveals two distinct classes of mRNA methylation at internal and 5' sites. Cell Rep 8, 284-296 (2014).

35. Wen, J. et al. Zc3h13 Regulates Nuclear RNA m6A Methylation and Mouse Embryonic Stem Cell Self-Renewal. Mol. Cell 69, 1028-1038.e6 (2018).

36. Liu, N. et al. N(6)-methyladenosine-dependent RNA structural switches regulate RNA-protein interactions. Nature 518, 560-564 (2015).

37. Luo, S. & Tong, L. Molecular basis for the recognition of methylated adenines in RNA by the eukaryotic YTH domain. Proc. Natl. Acad. Sci. U.S.A. 111, 13834-13839 (2014).

38. Xu, C. et al. Structural basis for selective binding of m6A RNA by the YTHDC1 YTH domain. Nat. Chem. Biol. 10, 927-929 (2014).

39. Zhu, T. et al. Crystal structure of the YTH domain of YTHDF2 reveals mechanism for recognition of N6-methyladenosine. Cell Res. 24, 1493-1496 (2014).

40. Xiao, W. et al. Nuclear m(6)A Reader YTHDC1 Regulates mRNA Splicing. Mol. Cell 61, 507-519 (2016).

41. Jia, G. et al. N6-methyladenosine in nuclear RNA is a major substrate of the obesity-associated FTO. Nat. Chem. Biol. 7, 885-887 (2011).

42. Gerken, T. et al. The obesity-associated FTO gene encodes a 2-oxoglutarate-dependent nucleic acid demethylase. Science 318, 1469-1472 (2007).

43. Sendinc, E. et al. PCIF1 Catalyzes m6Am mRNA Methylation to Regulate Gene Expression. Mol. Cell 75, 620-630.e9 (2019).

44. Wei, J. et al. Differential m6A, m6Am, and m1A Demethylation Mediated by FTO in the Cell Nucleus and Cytoplasm. Mol. Cell 71, 973-985.e5 (2018).

45. Li, Xiaoyu , et al. "Base-Resolution Mapping Reveals Distinct m(1)A Methylome in Nuclear- and Mitochondrial-Encoded Transcripts." Molecular Cell 68.5(2017).

46. Safra, M. et al. The m1A landscape on cytosolic and mitochondrial mRNA at single-base resolution. Nature 551, 251-255 (2017).

47. Dai, X., Wang, T., Gonzalez, G. & Wang, Y. Identification of YTH Domain-Containing Proteins as the Readers for N1-Methyladenosine in RNA. Anal. Chem. 90, 6380-6384 (2018).

48. Liu, F. et al. ALKBH1-Mediated tRNA Demethylation Regulates Translation. Cell 167, 1897 (2016).

49. Reid, R., Greene, P. J. & Santi, D. V. Exposition of a family of RNA m(5)C methyltransferases from searching genomic and proteomic sequences. Nucleic Acids Res. 27, 3138-3145 (1999).

50. Yang, X. et al. 5-methylcytosine promotes mRNA export - NSUN2 as the methyltransferase and ALYREF as an m5C reader. Cell Res. 27, 606-625 (2017).

51. Larrieu, D., Britton, S., Demir, M., Rodriguez, R. & Jackson, S. P. Chemical inhibition of NAT10 corrects defects of laminopathic cells. Science 344, 527-532 (2014).

52. Liu, X. et al. NAT10 regulates p53 activation through acetylating p53 at K120 and ubiquitinating Mdm2. EMBO Rep. 17, 349-366 (2016).

53. Ma, R. et al. Up regulation of NAT10 promotes metastasis of hepatocellular carcinoma cells through epithelial-to-mesenchymal transition. Am J Transl Res 8, 4215-4223 (2016).

54. Zhang, X. et al. N-Acetyltransferase 10 Enhances Doxorubicin Resistance in Human Hepatocellular Carcinoma Cell Lines by Promoting the Epithelial-to-Mesenchymal Transition. Oxid Med Cell Longev 2019, 7561879 (2019).

55. Sas-Chen, A. et al. Dynamic RNA acetylation revealed by quantitative cross-evolutionary mapping. Nature 583, 638-643 (2020).

56. Woo, H. H. & Chambers, S. K. Human ALKBH3-induced m1A demethylation increases the CSF-1 mRNA stability in breast and ovarian cancer cells. Biochim Biophys Acta Gene Regul Mech 1862, 35-46 (2019).

57. Blanco, S. et al. Stem cell function and stress response are controlled by protein synthesis. Nature 534, 335-340 (2016).

58. Wu, J., Zhu, H., Wu, J., Chen, W. & Guan, X. Inhibition of N-acetyltransferase 10 using remodelin attenuates doxorubicin resistance by reversing the epithelial-mesenchymal transition in breast cancer. Am J Transl Res 10, 256-264 (2018).

59. Szymańska, E., Markuszewski, M. J., Markuszewski, M. & Kaliszan, R. Altered levels of nucleoside metabolite profiles in urogenital tract cancer measured by capillary electrophoresis. J Pharm Biomed Anal 53, 1305-1312 (2010).

60. Li, H., Qin, Q., Shi, X., He, J. & Xu, G. Modified metabolites mapping by liquid chromatography-high resolution mass spectrometry using full scan/all ion fragmentation/neutral loss acquisition. J Chromatogr A 1583, 80-87 (2019).

61. Borek, E. et al. Altered excretion of modified nucleosides and beta-aminoisobutyric acid in subjects with acquired immunodeficiency syndrome or at risk for acquired immunodeficiency syndrome. Cancer Res. 46, 2557-2561 (1986).

62. Law, K. P., Han, T. L., Mao, X. & Zhang, H. Tryptophan and purine metabolites are consistently upregulated in the urinary metabolome of patients diagnosed with gestational diabetes mellitus throughout pregnancy: A longitudinal metabolomics study of Chinese pregnant women part 2. Clin. Chim. Acta 468, 126-139 (2017).

63. Niwa, T., Takeda, N. & Yoshizumi, H. RNA metabolism in uremic patients: accumulation of modified ribonucleosides in uremic serum. Technical note. Kidney Int. 53, 1801-1806 (1998).

64. Johansson, M. J. & Byström, A. S. The Saccharomyces cerevisiae TAN1 gene is required for N4-acetylcytidine formation in tRNA. RNA 10, 712-719 (2004).

65. Thomas, G., Gordon, J. & Rogg, H. N4-Acetylcytidine. A previously unidentified labile component of the small subunit of eukaryotic ribosomes. J. Biol. Chem. 253, 1101-1105 (1978).

66. Sharma, S. et al. Yeast Kre33 and human NAT10 are conserved 18S rRNA cytosine acetyltransferases that modify tRNAs assisted by the adaptor Tan1/THUMPD1. Nucleic Acids Res. 43, 2242-2258 (2015).

67. Li, X. et al. Transcriptome-wide mapping reveals reversible and dynamic N(1)-methyladenosine methylome. Nat. Chem. Biol. 12, 311-316 (2016).

68. Alarcón, C. R. et al. HNRNPA2B1 Is a Mediator of m(6)A-Dependent Nuclear RNA Processing Events. Cell 162, 1299-1308 (2015).

69. Zeltz, C. et al. Cancer-associated fibroblasts in desmoplastic tumors: emerging role of integrins. Semin. Cancer Biol. 62, 166-181 (2020).

70. Guan, X. Cancer metastases: challenges and opportunities. Acta Pharm Sin B 5, 402-418 (2015).

71. Levental, K. R. et al. Matrix crosslinking forces tumor progression by enhancing integrin signaling. Cell 139, 891-906 (2009).

72. Egeblad, M., Rasch, M. G. & Weaver, V. M. Dynamic interplay between the collagen scaffold and tumor evolution. Curr. Opin. Cell Biol. 22, 697-706 (2010).

73. Reig, G., Pulgar, E. & Concha, M. L. Cell migration: from tissue culture to embryos. Development 141, 1999-2013 (2014).

74. Löfberg, J., Ahlfors, K. & Fällström, C. Neural crest cell migration in relation to extracellular matrix organization in the embryonic axolotl trunk. Dev. Biol. 75, 148-167 (1980).

Figure. S1.


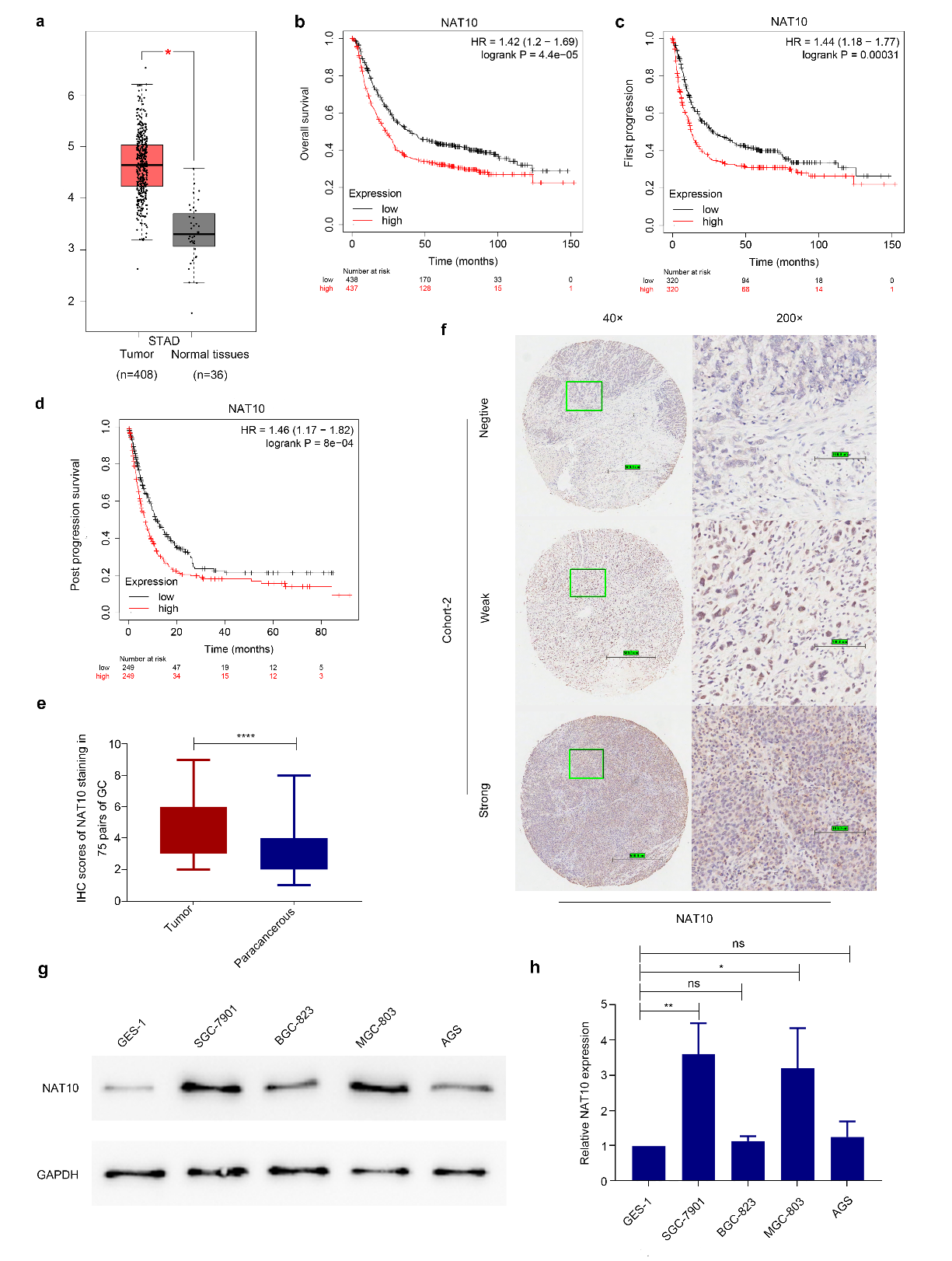


**Figure. S1 a.** The expression of NAT10 in the TCGA STAD dataset. **b-d.** High NAT10 expression was significantly associated with a shorter OS, PPS, and FP. **e.** IHC scores of NAT10 staining in 75 pairs of GC tissues and matching normal tissues, determined via immunohistochemical staining intensity finally score method. **f.** Representative images of IHC staining for the NAT10 protein on a TAM constructed from 75 GC tissues. **g.** Western blotting analysis of the NAT10 in different cell lines. **h.** qRT-PCR analysis of NAT10 in different cell lines.

Figure. S2.


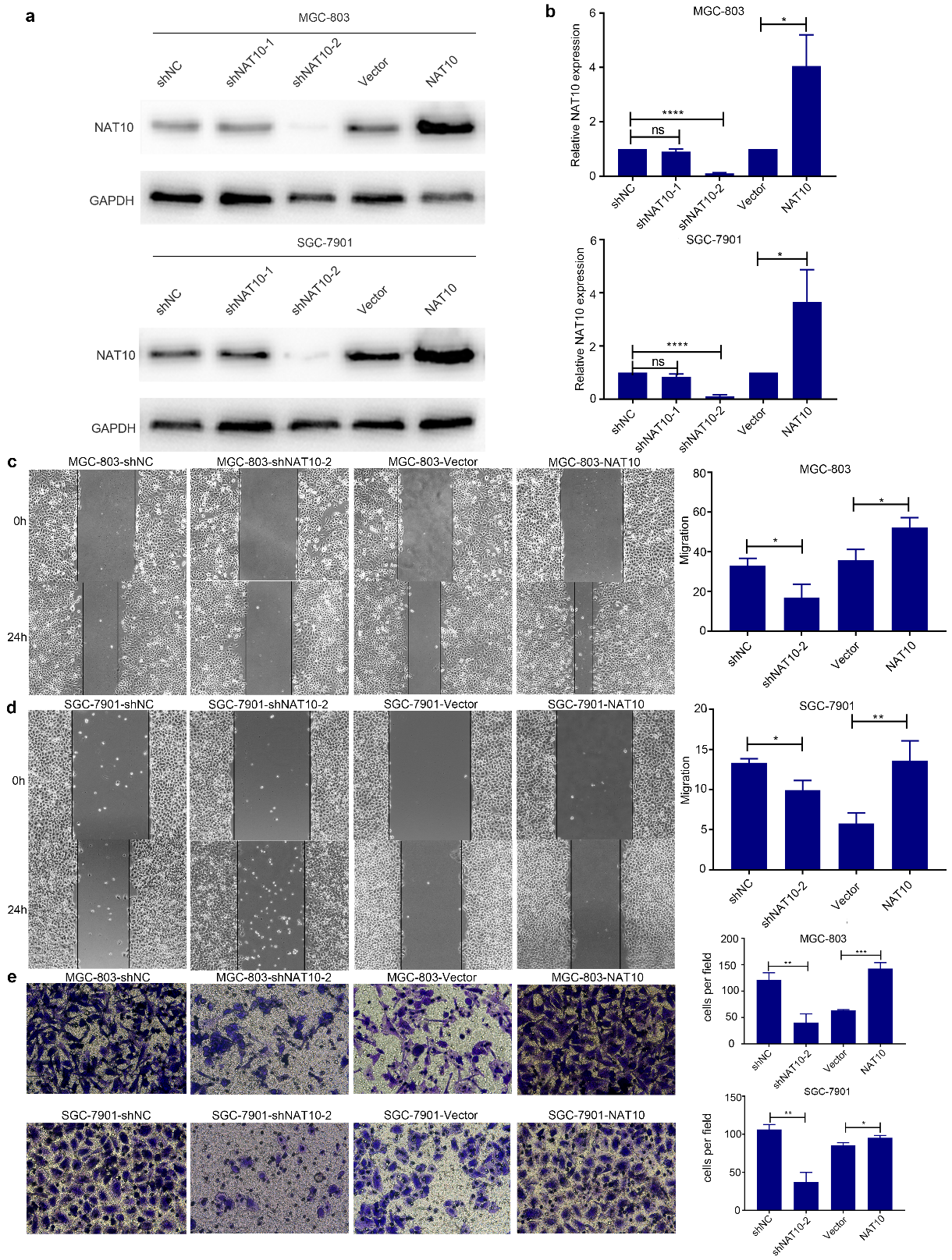


**Figure. S2 a.** The shRNA-mediated NAT10 repression and NAT10 overexpression confirmed by western blotting assay after lentivirus infection in the MGC-803 and SGC-7901 cells. **b.** The shRNA-mediated NAT10 repression and NAT10 overexpression confirmed by qRT-PCR assay after lentivirus infection in the MGC-803 and SGC-7901 cells. **c-d.** The wound healing of NAT10 knockdown or overexpression for 24h were recorded (left) and quantitatively analyzed (right). **e.** The knockdown or overexpression of NAT10 cell invasion ability was recorded (left) and quantitatively analyzed (right) by transwell matrigel invasion assay.

Figure. S3.


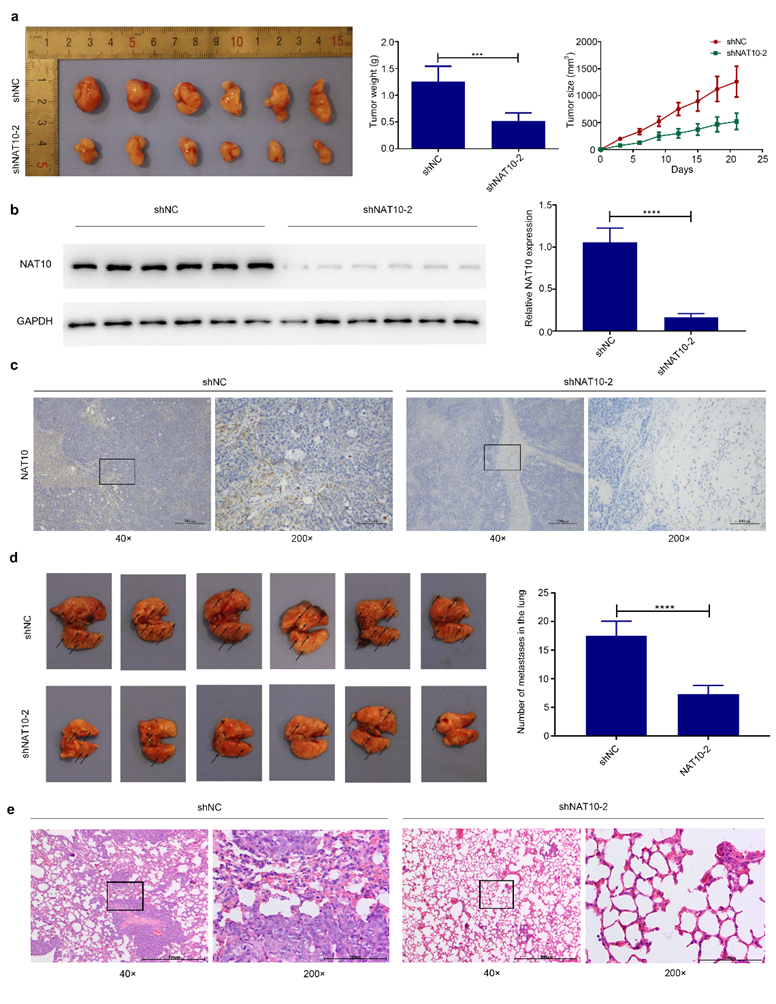


**Figure. S3 a.** Knockdown of NAT10 effectively suppressed GC subcutaneous tumor growth in nude mice, and the tumor weight was quantitatively analyzed. The size of the tumor formed in the subcutaneous implantation mice model was monitored every 3 days. **b.** The expression of NAT10 in the subcutaneous tumor was confirmed by western blotting and qRT-PCR analyses. **c.** IHC (NAT10)-stained paraffin-embedded sections obtained from the sh-NC and shNAT10-2 SGC-7901 subcutaneous tumors. **d.** SGC-7901-shNC and SGC-7901-shNAT10 stable cells were injected into the nude mice via the tail vein. Representative images of metastatic lung tumors are shown (left) and the number of lung tumors was quantitatively analyzed (right). **e.** H&E staining results of the metastatic lung tumors are shown.

Figure. S4.

**
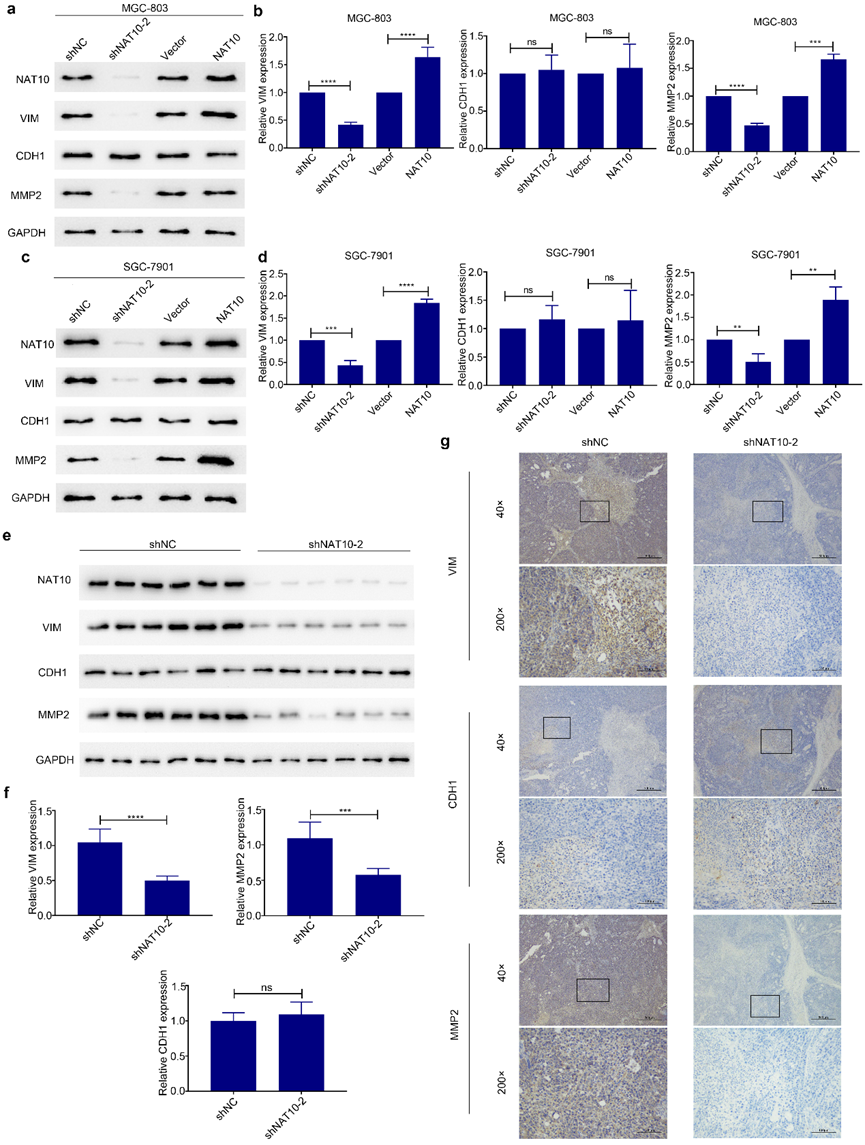
**

**Figure. S4 a-b.** Western blotting and qRT-PCR analyses of NAT10 and EMT markers in MGC-803. **c-d.** Western blotting and qRT-PCR analyses of NAT10 and EMT markers in SGC-7901. **e-f.** Western blotting and qRT-PCR analyses of NAT10 and EMT markers in tumor tissues obtained from the sh-NC and shNAT10-2 SGC-7901 subcutaneous tumor **g.** IHC (VIM, CDH1, and MMP2)-stained paraffin-embedded sections obtained from the sh-NC and shNAT10-2 SGC-7901 subcutaneous tumors.

Figure. S5.

**
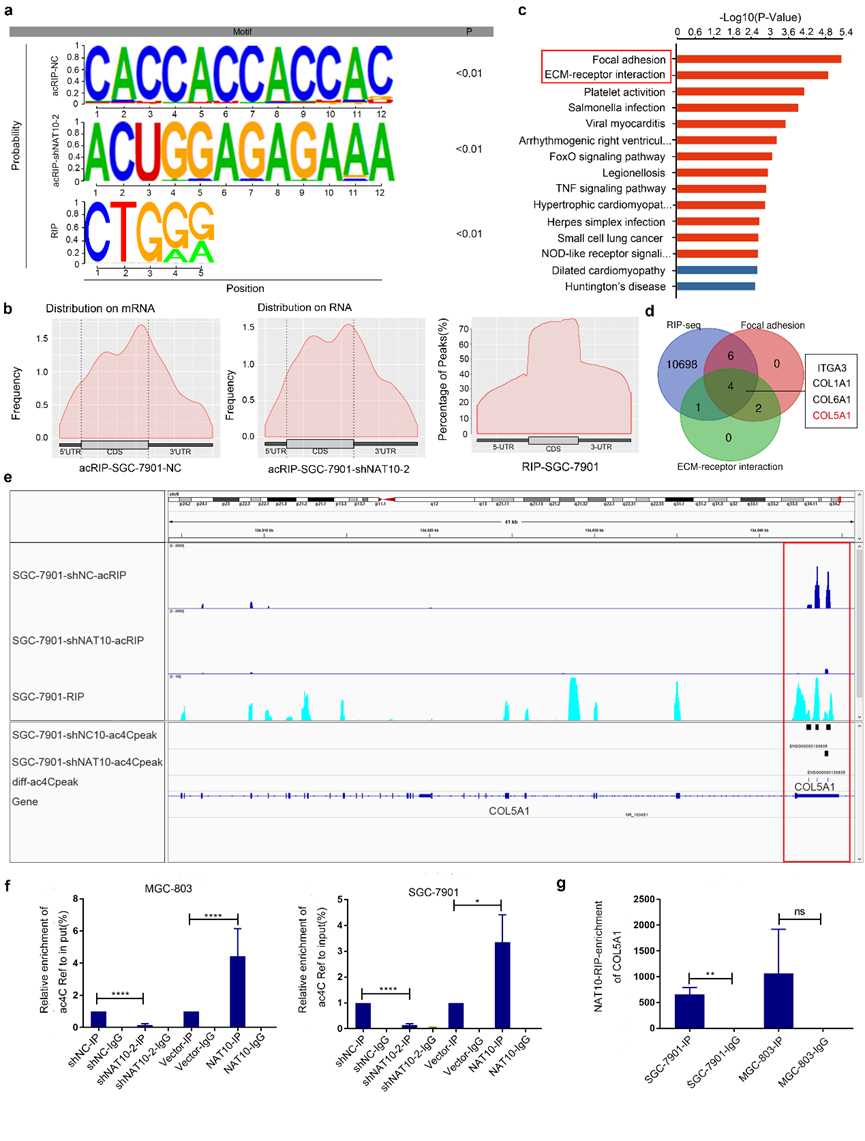
**

**Figure. S5 a.** The ac4C significant consensus sequence motif was identified based on the acRIP-seq and RIP-seq analyses, and the significant consensus sequence of NAT10 bound and interacted. **b.** Frequency distribution of the ac4C peaks across the mRNA transcripts based on the acRIP-seq and RIP-seq analyses, and the distribution of NAT10 bound region. **c.** KEGG pathway enrichment analysis and acRIP-seq analysis identified the enriched pathway of ac4C modification down-regulated genes after NAT10 silencing. **d.** Overlapping the potential target bound and interacted with NAT10 in focal adhesion and ECM-receptor interaction pathway by the acRIP-seq and RIP-seq analysis. **e.** Attenuation of the NAT10 diminishes the ac4C modification genome of COL5A1 mRNA visual result compared using the acRIP-seq (colored in deep blue). The visual genome result of COL5A1 mRNA bound and interacted with NAT10 (colored in wathet blue). **f.** The regulatory role of NAT10 on COL5A1 ac4C in SGC-7901 and MGC-803 cells confirmed by the acRIP-qPCR assay. **g.** The bound and interacted relationship between NAT10 and COL5A1 mRNA in SGC-7901 and MGC-803 cells confirmed using the RIP-qPCR assay.

Figure. S6.

**
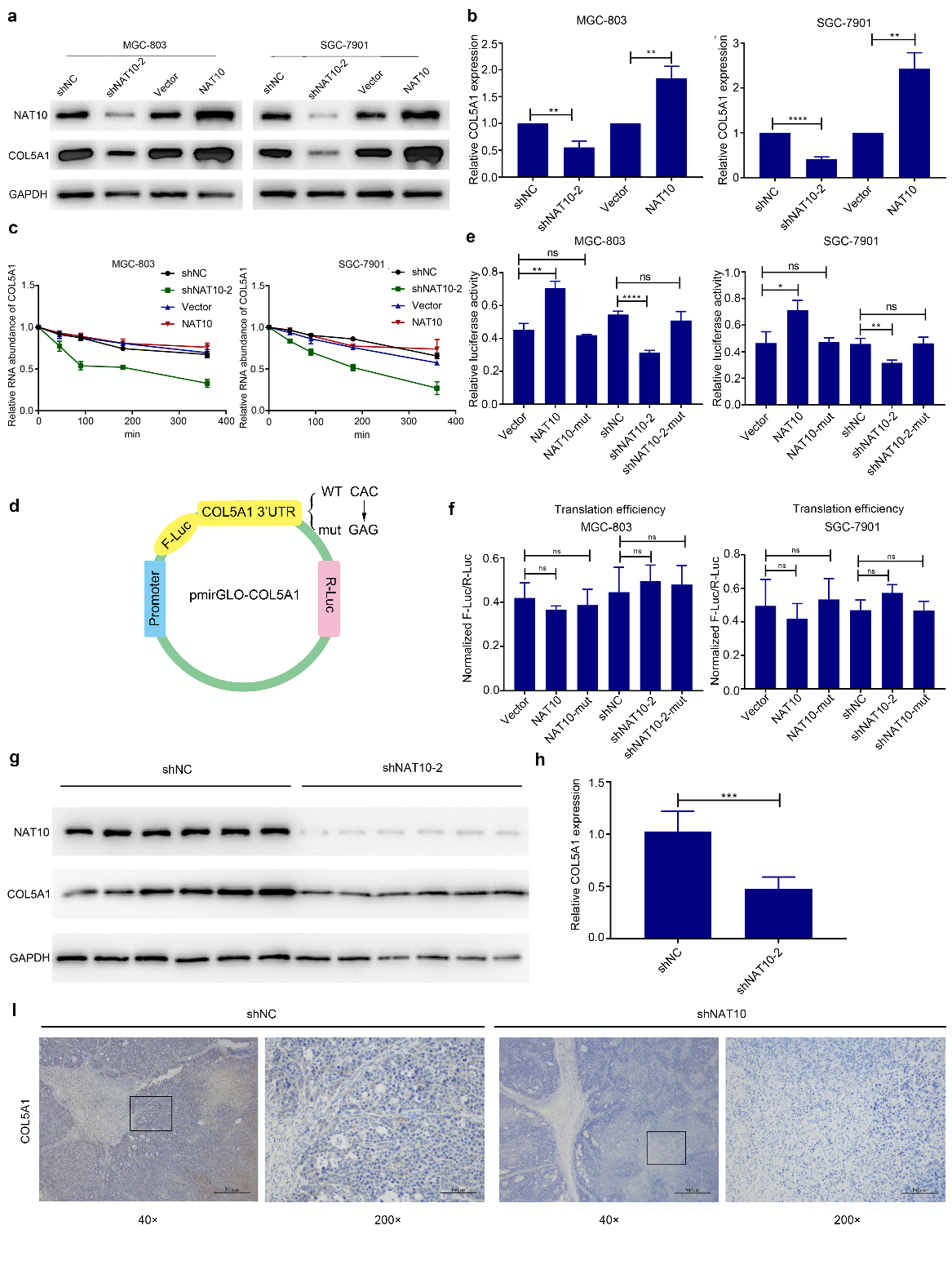
**

**Figure. S6 a-b.** Western blotting and qRT-PCR analyses of COL5A1 in shNAT10 or NAT10 overexpressing MGC-803 and SGC-7901 cells. **c.** The impression of NAT10 on COL5A1 mRNA stability confirmed by the RNA decay assay. **d.** Wild-type (WT) or mutant COL5A1 cells were transfected with pmirGLO-COL5A1 reporter, respectively. **e.** The transcriptional level of wild-type COL5A1, but not the mutation, significantly decreased in the NAT10-knockdown cells, and significantly increased in the NAT10 overexpressing cells. **f.** The impression of NAT10 on COL5A1 mRNA translation efficiency confirmed. **g-i.** The expression of COL5A1 was confirmed by western blotting, qRT-PCR analyses, and IHC in shNC and shNAT10-2 SGC-7901 subcutaneous tumors.

Figure. S7.

**
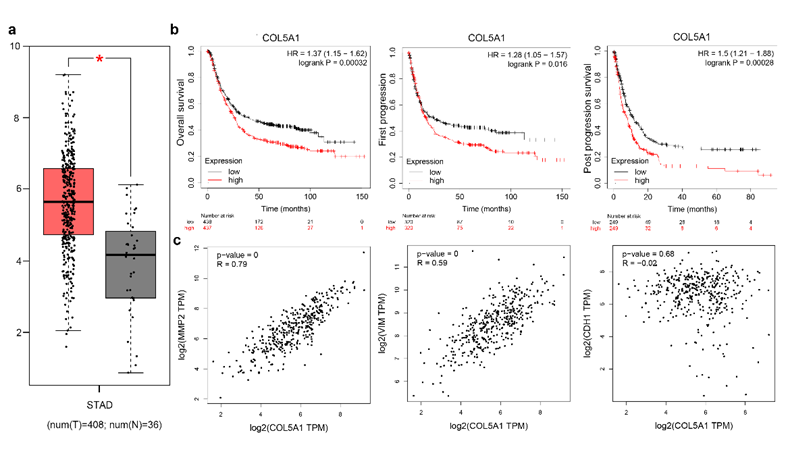
**

**Figure. S7 a.** The expression of COL5A1 in the TCGA STAD dataset. **b.** High COL5A1 expression was significantly associated with a shorter OS, PPS and FP. **c.** The correlation between COL5A1 with MMP2, VIM, and CDH1 expression in TCGA.

Figure. S8.


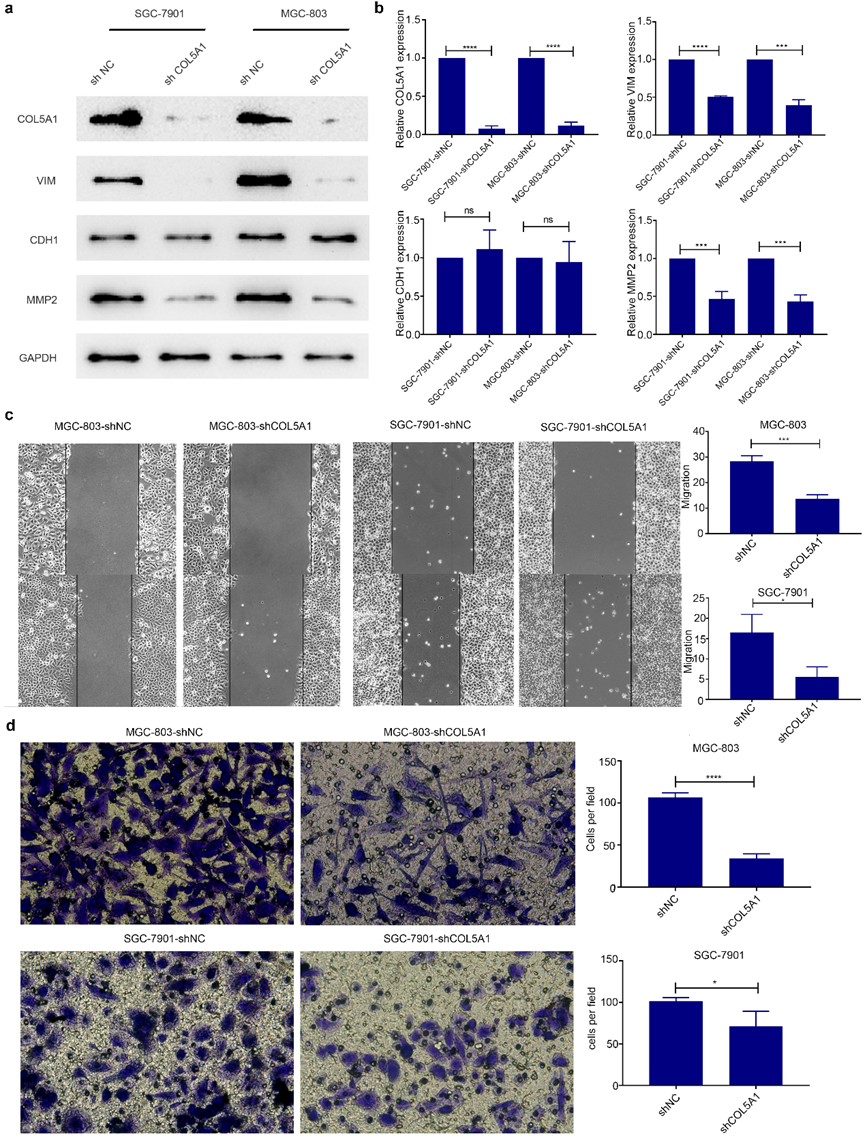


**Figure. S8 a-b.** Western blot and qRT-PCR analyses of COL5A1 and EMT markers in the sh-NC, shCOL5A1 MGC-803, and SGC-7901 cells. **c.** The wound healing of COL5A1 knockdown in the MGC-803 and SGC-7901 for 24h were recorded (left) and quantitatively analyzed (right). **d.** The knockdown or overexpression of COL5A1 in MGC-803 and SGC-7901 were recorded (left) and quantitatively analyzed (right) by transwell matrigel invasion assay.

Figure. S9.


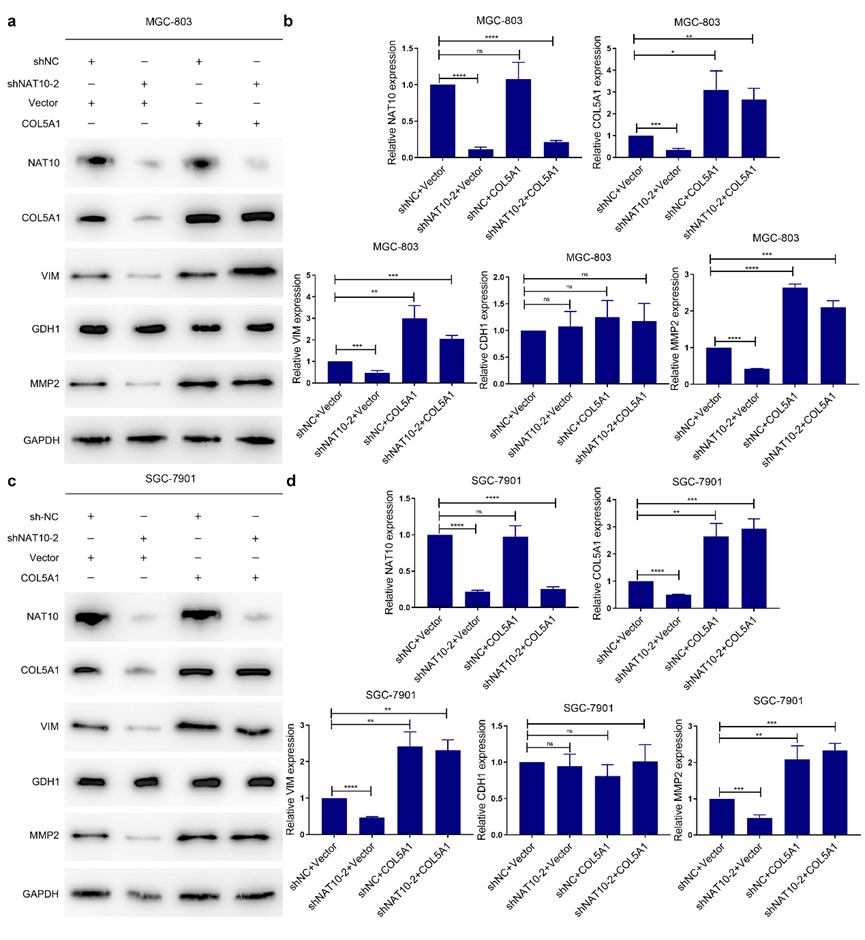


**Figure. S9 a-b.** The NAT10 and EMT marker protein and mRNA levels were examined by western blotting and qRT-PCR analyses in the MGC-803 cells transfected with lentiviruses containing COL5A1 and/or shNAT10. **c-d.** NAT10 and EMT marker protein and mRNA levels were examined by western blot and qRT-PCR analyses in the SGC-7901 cells transfected with lentiviruses carrying COL5A1 and/or shNAT10.

Figure. S10.


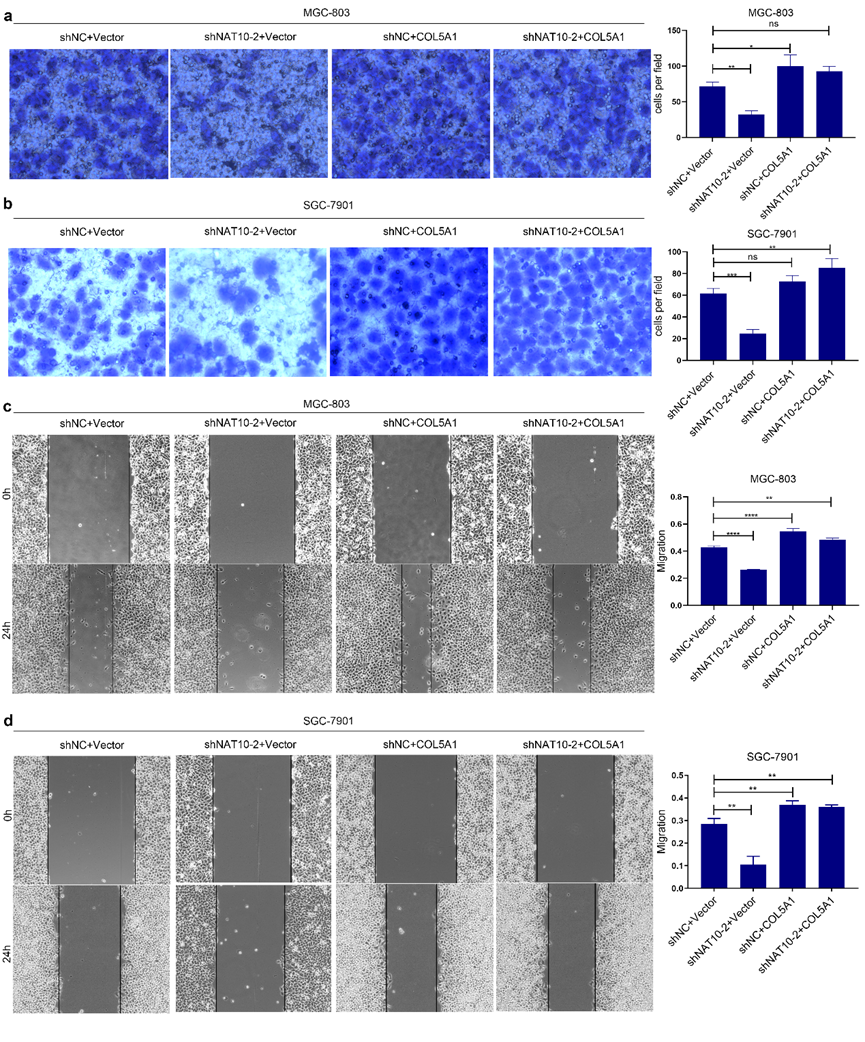


**Figure. S10 a-b.** The invasive abilities were explored by transwell matrigel invasion assay in the MGC-803 and SGC-7901 cells transfected with lentiviruses carrying COL5A1 and/or shNAT10 and quantitatively analyzed (right). **c-d.** The migration abilities were explored by wound healing assay in the MGC-803 and SGC-7901 cells transfected with lentiviruses carrying COL5A1 and/or shNAT10 and quantitatively analyzed (right).

Figure. S11.


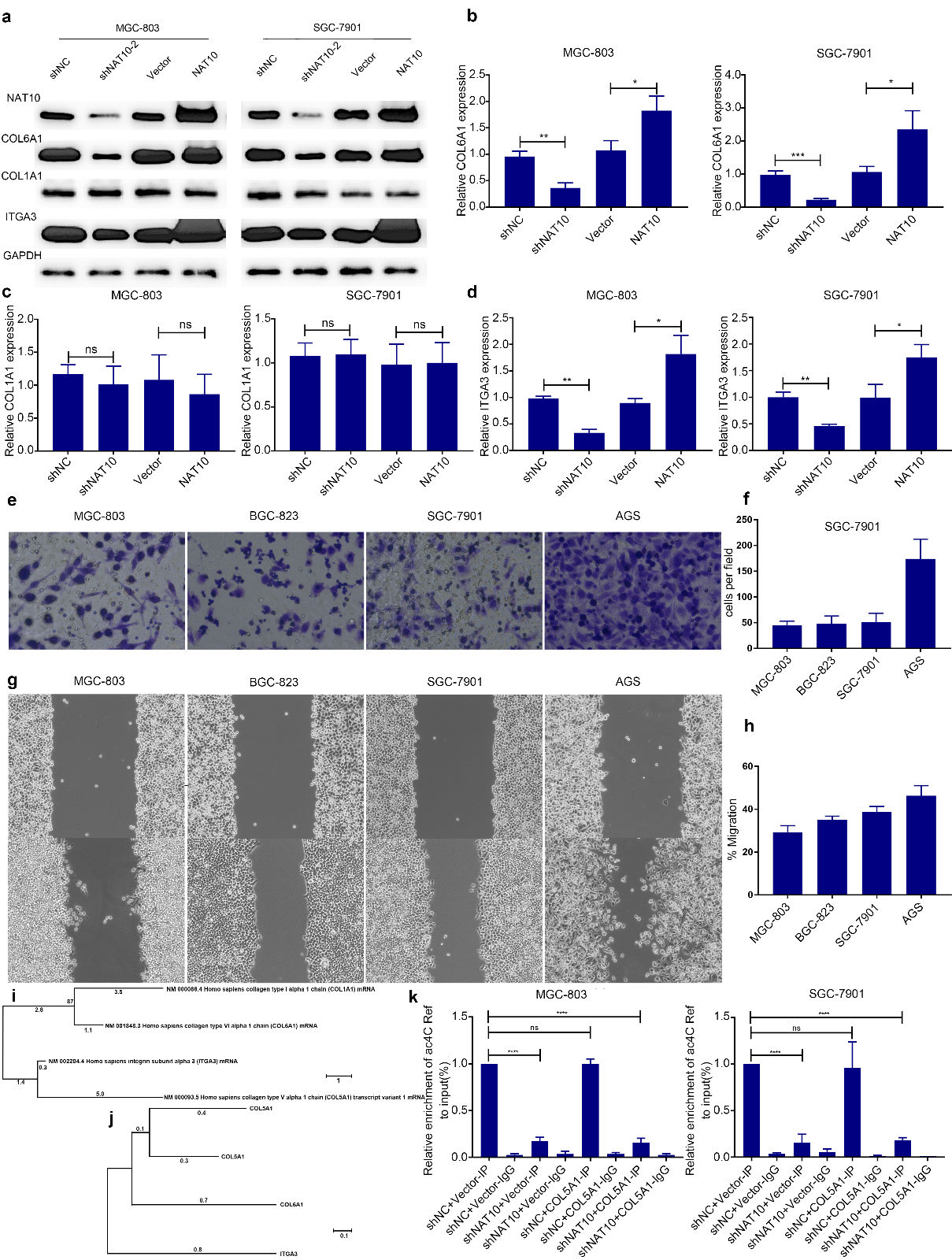
­­

**Figure. S11 a-d.** Western blotting and qRT-PCR analyses of COL1A1, COL1A1 and ITGA3 in shNAT10 or NAT10 overexpressing MGC-803 and SGC-7901 cells. **e-f.** The invasive abilities were explored by transwell matrigel invasion assay in the MGC-803, BGC-823, SGC-7901 and AGS cells and quantitatively analyzed (right). **g-h**. The migration abilities were explored by wound healing assay in the MGC-803, BGC-823, SGC-7901 and AGS cells and quantitatively analyzed (right). **i.** The homology analysis of COL5A1\COL1A1\COL6A1\ITGA3 based on mRNA sequence. **j.** The homology analysis of COL5A1\COL1A1\COL6A1\ITGA3 based on DNA sequence. **k.** ac4C modification on COL5A1 mRNA confirmed by the acRIP-qPCR assay.

Figure. S12.


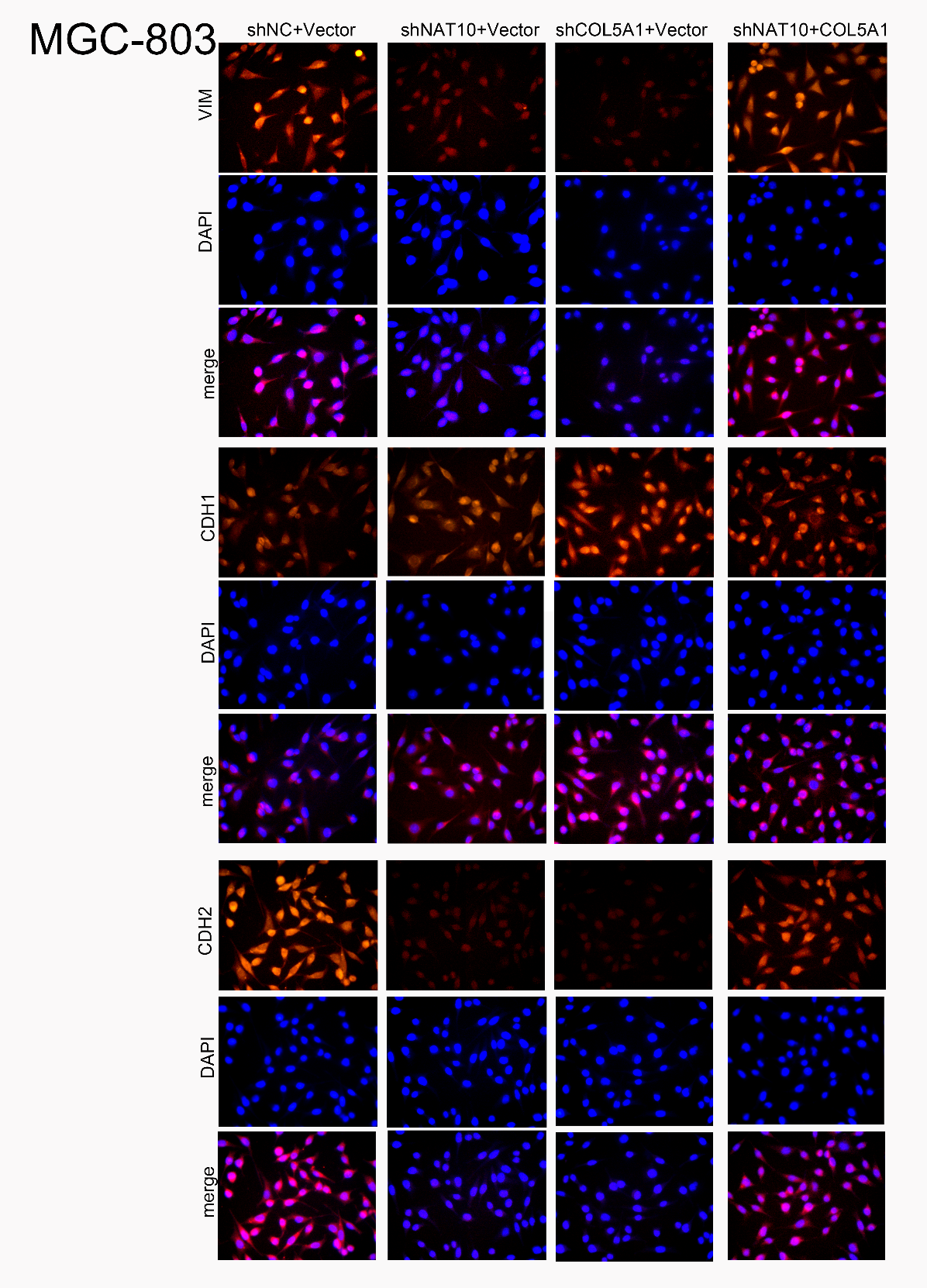


**Figure. S12** The EMT marker protein levels were examined by Immunofluorescence in the MGC-803 cells transfected with lentiviruses, containing COL5A1 and/or shNAT10 (400X).

Figure. S13.


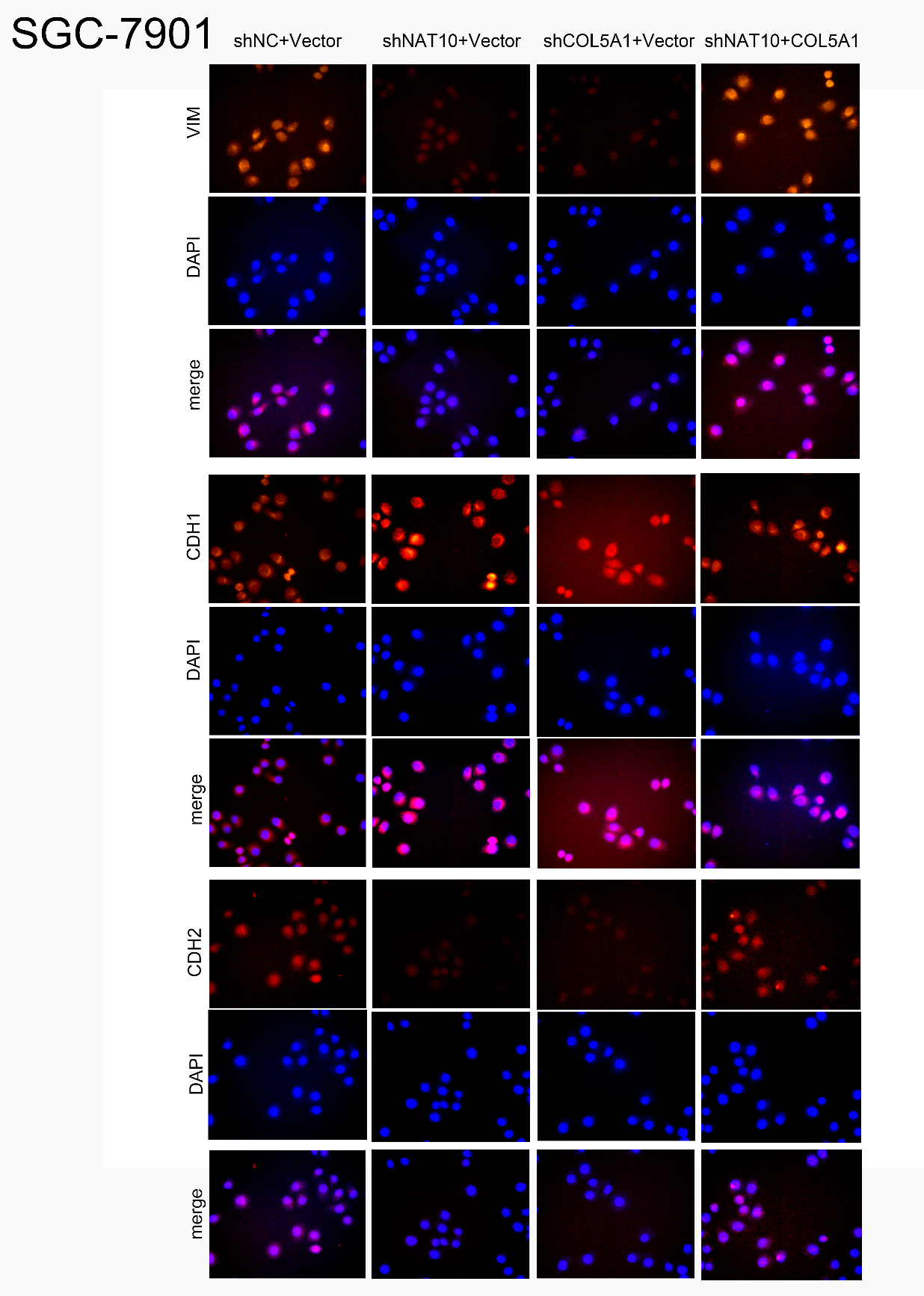


**Figure. S13** The EMT marker protein levels were examined by Immunofluorescence in the SGC-7901 cells transfected with lentiviruses, containing COL5A1 and/or shNAT10 (400X).

Figure. S14.


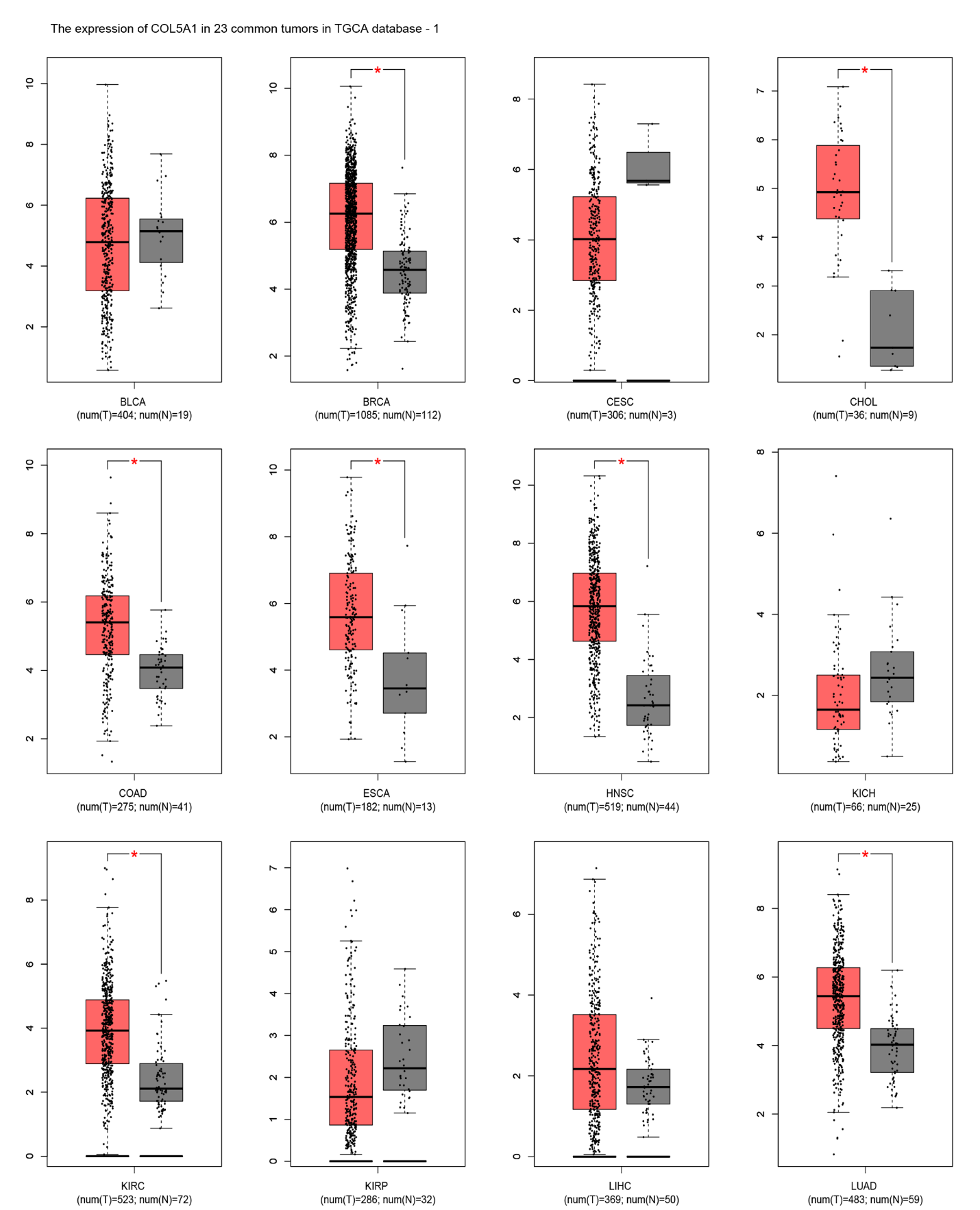


**Figure S14.** The expression of COL5A1 in 23 common tumors in TGCA database, the expression levels of NAT10 was significantly up-regulated in STAD, CHOL, READ and COAD.

Figure. S15.


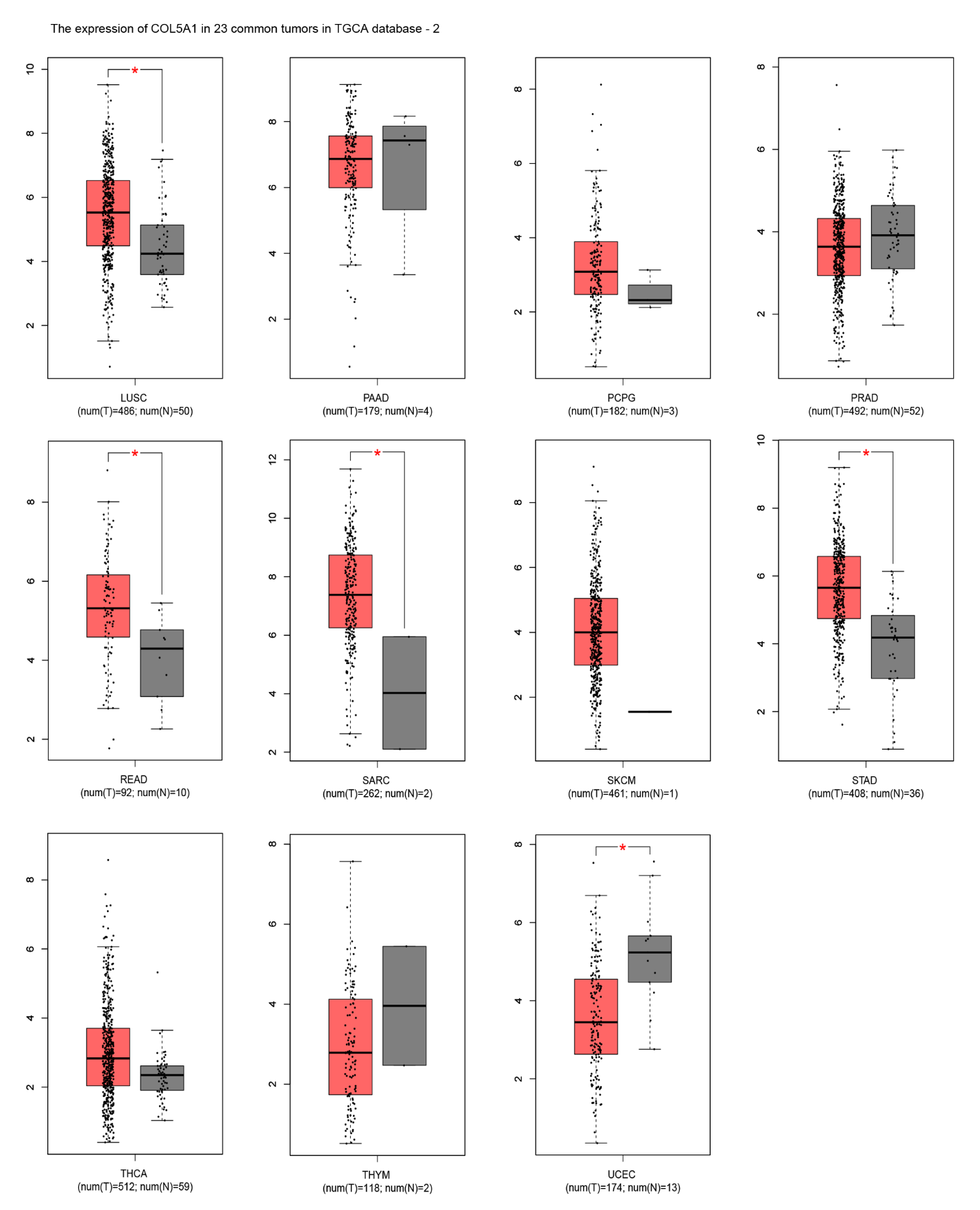


**Figure S15.** The expression of COL5A1 in 23 common tumors in TGCA database, the expression levels of NAT10 was significantly up-regulated in STAD, CHOL, READ and COAD.

Figure. S16.


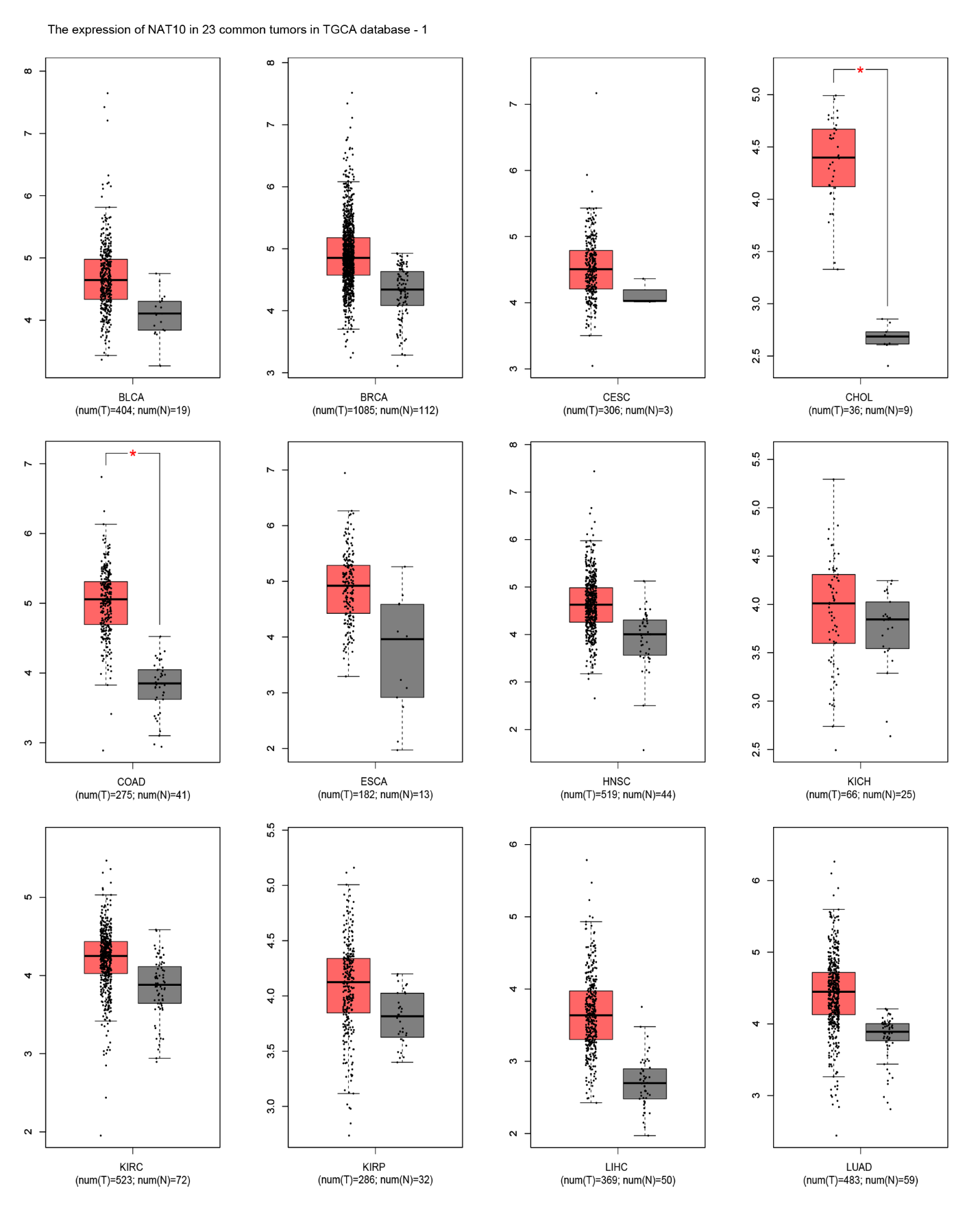


**Figure S16.** The expression of NAT10 in 23 common tumors in TGCA database, the expression levels of NAT10 was significantly up-regulated in STAD, CHOL, READ and COAD.

Figure. S17.


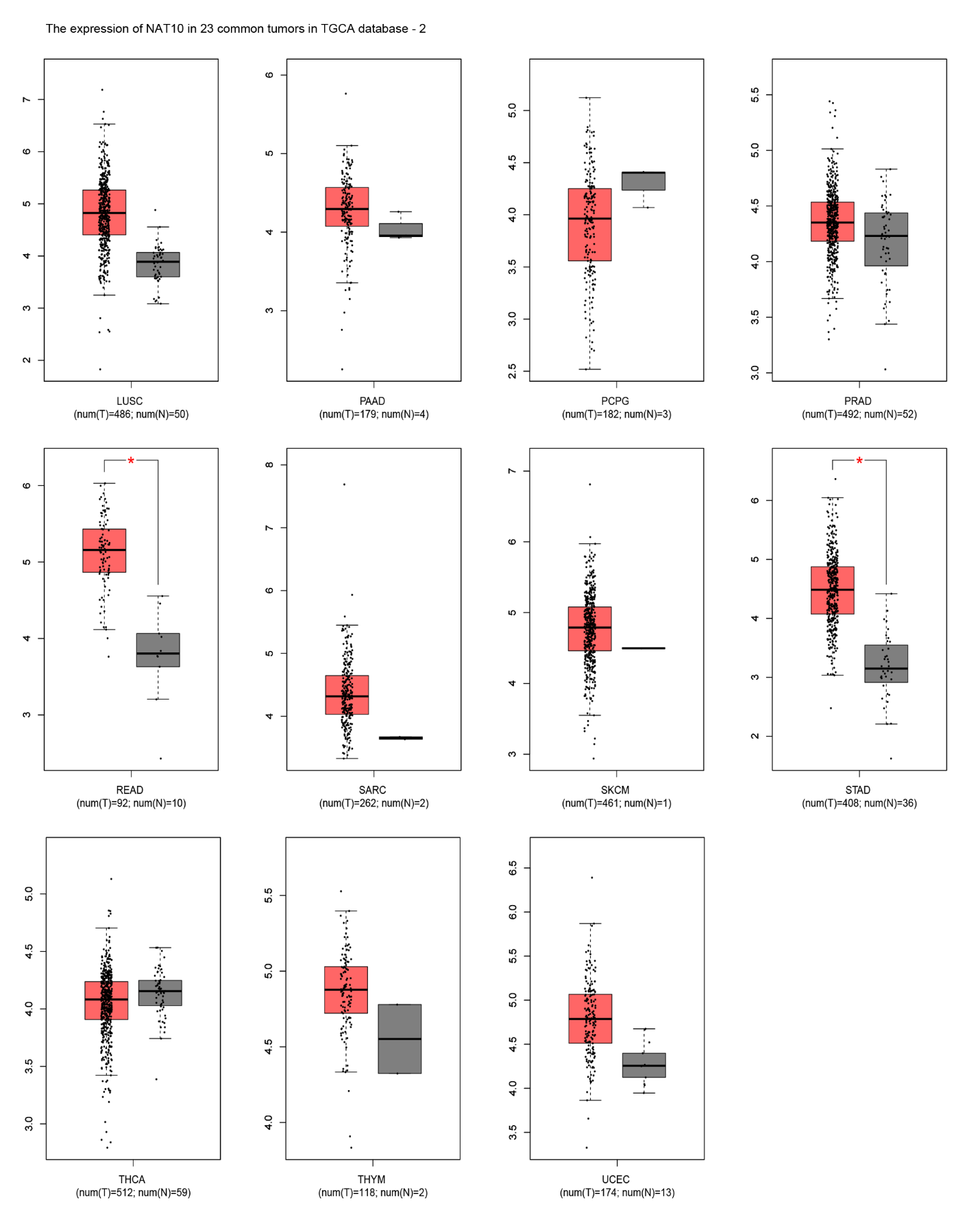


**Figure S17.** The expression of NAT10 in 23 common tumors in TGCA database, the expression levels of NAT10 was significantly up-regulated in STAD, CHOL, READ and COAD.

Figure. S18.


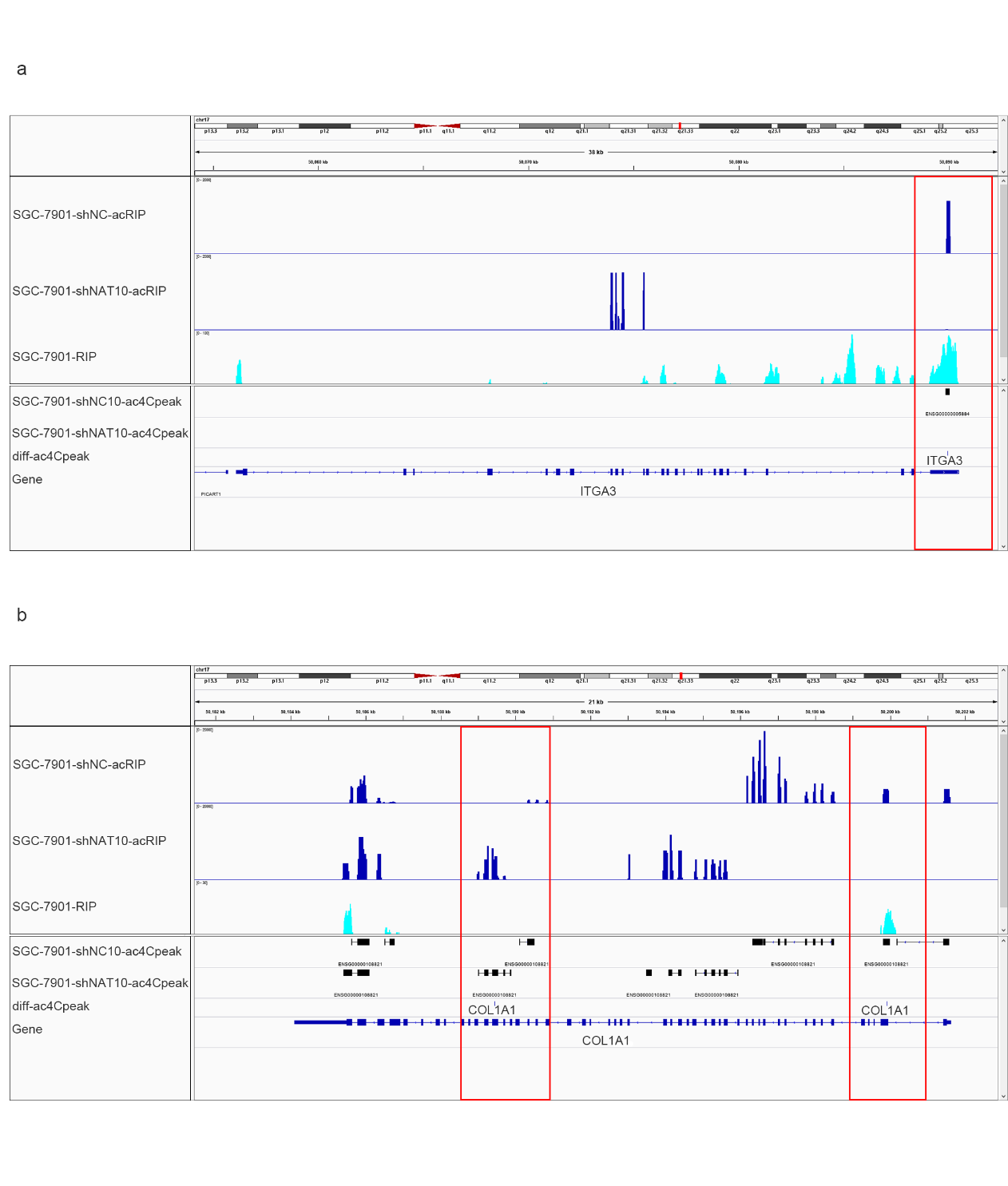


Figure. S18. Attenuation of NAT10 diminished ac4C modification of selected 4 mRNAs in SGC-7901. The annotation in the box of SGC-7901-shNC-ac4C PEAK represents the distribution of ac4C PEAK at the location of the selected gene in SGC-7901-shNC cells. The annotation in the box of SGC-7901-shNAT10-ac4C PEAK represents the distribution of ac4C PEAK at the location of the selected gene in SGC-7901-shNAT10 cells. diff -ac4C Peak represents that the ac4C distribution of this position changes after NAT10 expression is down-regulated a. Attenuation of NAT10 diminished ac4C modification of ITGA3 in SGC-7901. b. Attenuation of NAT10 diminished ac4C modification of COL1A1 in SGC-7901.

Figure. S19.


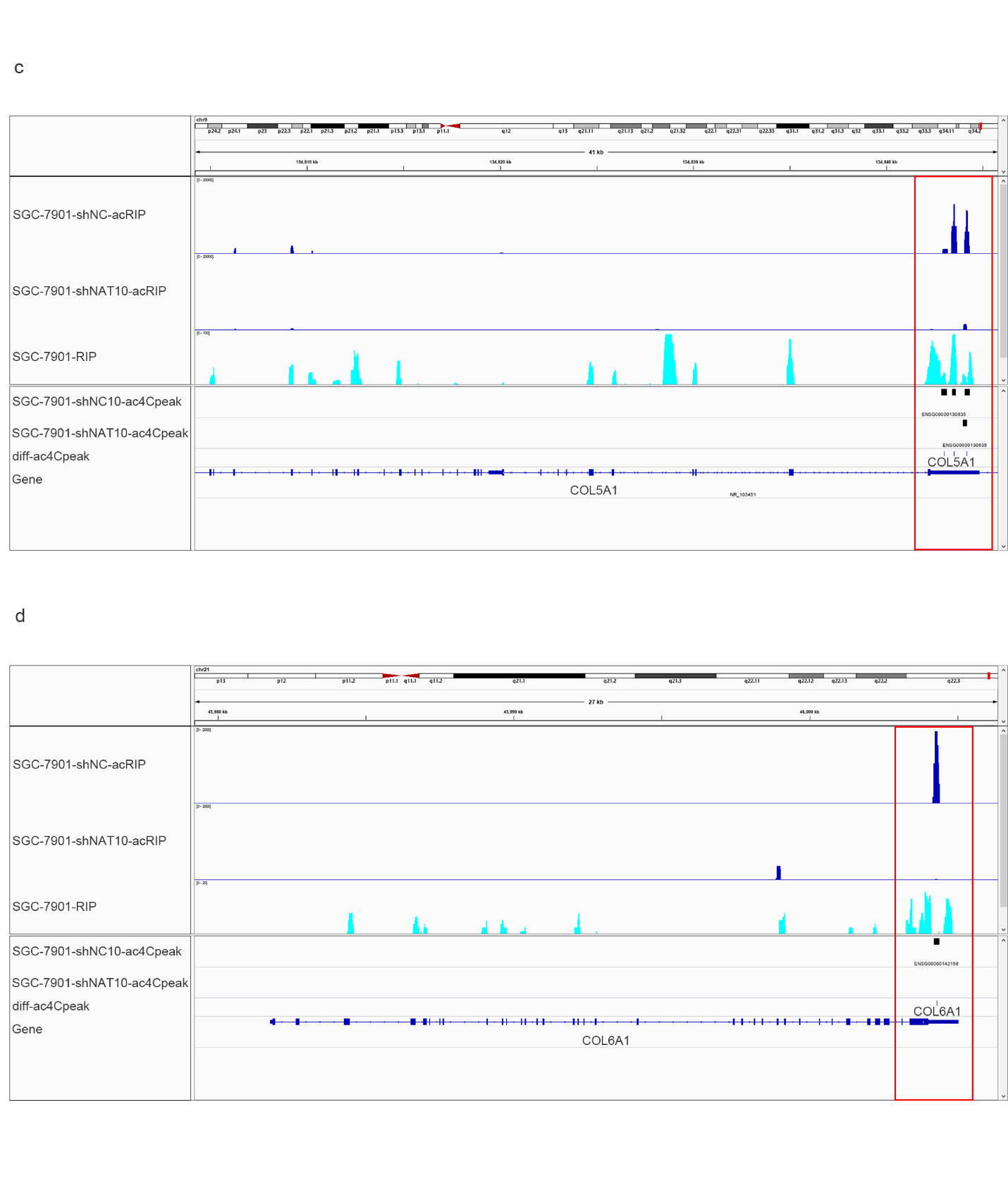


**Figure. S19 c.** Attenuation of NAT10 diminished ac4C modification of COL5A1 in SGC-7901. **d.** Attenuation of NAT10 diminished ac4C modification of COL6A1 in SGC-7901.

Table S1.


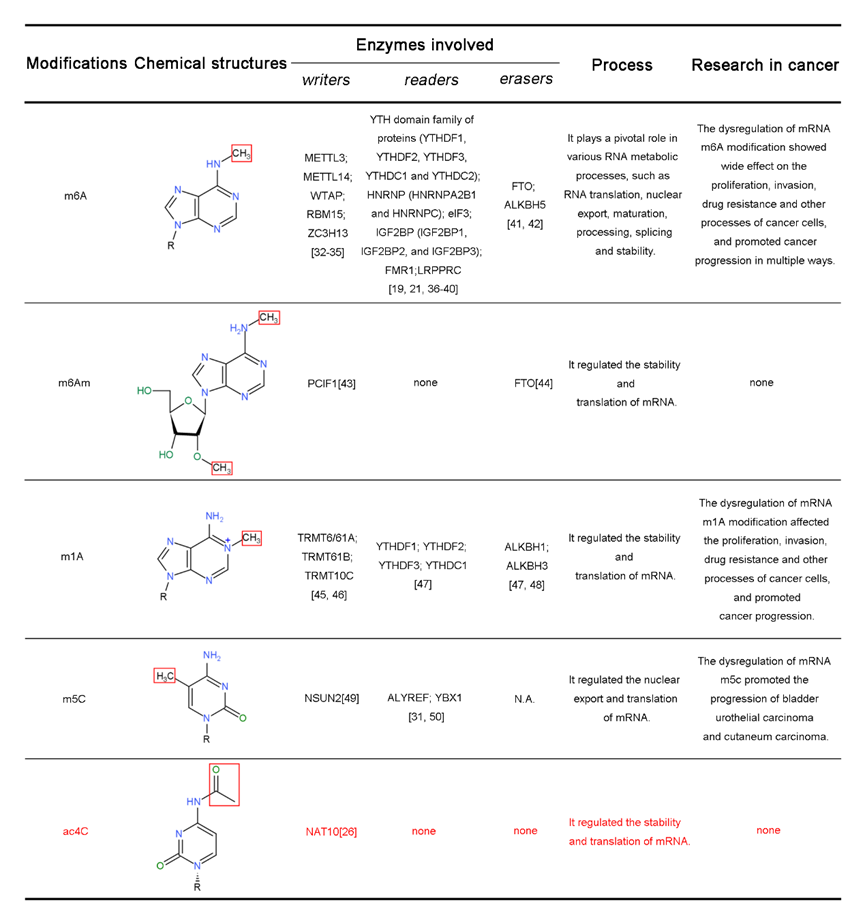


Table S2. Association between NAT0 expression and clinicopathological characteristics in gastric cancer

| Characteristics | NAT10 expression | | n | x^2^ | *p* value |
| --- | --- | --- | --- | --- | --- |
|  | Negative | Strong |  |  |  |
| Gender |  |  |  |  |  |
| Female | 9 | 7 | 16 | 1.239 | 0.266 |
| Male | 24 | 35 | 59 |  |  |
| Age (Years) |  |  |  |  |  |
| ≥55 | 23 | 34 | 57 | 0.012 | 0.912 |
| <55 | 7 | 11 | 18 |  |  |
| Histopathologic types |  |  |  |  |  |
| Gastric cancer tissue | 33 | 42 | 75 | 24.144 | ＜0.0001 |
| Normal tissue | 62 | 13 | 75 |  |  |
| Pathologic grading |  |  |  |  |  |
| Ⅱ | 11 | 13 | 24 | 0.048 | 0.826 |
| Ⅲ | 22 | 29 | 51 |  |  |
| Location |  |  |  |  |  |
| Other | 27 | 31 | 58 | 0.676 | 0.411 |
| Cardia | 6 | 11 | 17 |  |  |
| Lymph vessel invasion |  |  |  |  |  |
| No | 17 | 8 | 25 | 8.766 | 0.003 |
| Yes | 16 | 34 | 50 |  |  |
| pT stage |  |  |  |  |  |
| T1  T2  T3  T4 | 2 | 3 | 5 |  | 0.273 |
|  | 6 | 3 | 9 |  |  |
|  | 20 | 23 | 43 |  |  |
|  | 5 | 13 | 18 |  |  |
| pN stage |  |  |  |  |  |
| N0  N1  N2  N3 | 17 | 8 | 25 |  | 0.069 |
|  | 7 | 8 | 15 |  |  |
|  | 6 | 8 | 14 |  |  |
|  | 3 | 18 | 21 |  |  |
| pM stage |  |  |  |  |  |
| M0  M1 | 32 | 35 | 67 |  | 0.058 |
|  | 1 | 7 | 8 |  |  |
| ACJJ |  |  |  |  |  |
| Ⅰ  Ⅱ  Ⅲ  Ⅳ | 4 | 3 | 7 |  | 0.009 |
|  | 20 | 11 | 31 |  |  |
|  | 8 | 21 | 29 |  |  |
|  | 1 | 7 | 8 |  |  |

Table S3. shRNA and primer sequences

| sh-NAT10-1 | TTGCTGTTCACCCAGATTATCCTCGAGGATAATCTGGGTGAACAGCAA |
| --- | --- |
| sh-NAT10-2 | GAGATGTATTCACGGAATATGCTCGAGCATATTCCGTGAATACATCTC |
| sh-COL5A1 | CCAGATTTGGACACTATATTTCTCGAGAAATATAGTGTCCAAATCTGG |
| NAT10 forward primer | ATAGCAGCCACAAACATTCGC |
| NAT10 reverse primer | ACACACATGCCGAAGGTATTG |
| COL5A1 forward primer | GCCCGGATGTCGCTTACAG |
| COL5A1 reverse primer | AAATGCAGACGCAGGGTACAG |
| VIM forward primer | GACGCCATCAACACCGAGTT |
| VIM reverse primer | CTTTGTCGTTGGTTAGCTGGT |
| MMP2 forward primer | TACAGGATCATTGGCTACACACC |
| MMP2 reverse primer | GGTCACATCGCTCCAGACT |
| CDH1 forward primer | CGAGAGCTACACGTTCACGG |
| CDH1 reverse primer | GGGTGTCGAGGGAAAAATAGG |
| COL1A1 forward primer | GAGGGCCAAGACGAAGACATC |
| COL1A1 reverse primer | CAGATCACGTCATCGCACAAC |
| COL6A1 forward primer | ACAGTGACGAGGTGGAGATCA |
| COL6A1 reverse primer | GATAGCGCAGTCGGTGTAGG |
| ITGA3 forward primer | TCAACCTGGATACCCGATTCC |
| ITGA3 reverse primer | GCTCTGTCTGCCGATGGAG |
| β-actin forward primer | CTCCATCCTGGCCTCGCTGT |
| β-actin reverse primer | GCTGTCACCTTCACCGTTCC |
| 18S rRNA forward primer | CAGCCACCCGAGATTGAGCA |
| 18S rRNA reverse primer | TAGTAGCGACGGGCGGTGTG |
| acRIP forward primer | 5'TCCTGACCCTGGACAGTGAAGGC 3' |
| acRIP reverse primer | 5'TGGTGCCGAGGCGTAGATGAAGT 3' |
| RIP forward primer | 5'TCCTGACCCTGGACAGTGAAGGC 3' |
| RIP reverse primer | 5' TGGTGCCGAGGCGTAGATGAAGT3' |

Table S4. Overexpression RNA and 3’UTR sequences.

| OE-NAT10 | ATGCATCGGAAAAAGGTGGATAACCGAATCCGGATTCTCATTGAGAATGGAGTAGCTGAGCGGCAAAGATCTCTCTTTGTTGTAGTTGGGGATCGAGGAAAAGATCAGGTGGTAATACTTCATCACATGTTATCCAAAGCAACTGTGAAGGCTCGGCCTTCAGTGCTGTGGTGTTATAAGAAAGAGCTGGGGTTTAGCAGTCACCGGAAGAAAAGAATGCGACAGCTGCAGAAGAAAATAAAGAATGGAACACTGAACATAAAGCAGGACGACCCCTTTGAACTCTTCATAGCAGCCACAAACATTCGCTACTGCTACTACAACGAGACCCACAAGATCCTGGGCAATACCTTCGGCATGTGTGTGCTGCAGGATTTTGAAGCCTTAACTCCAAACTTGCTGGCCAGGACTGTAGAAACAGTGGAAGGTGGTGGGCTAGTGGTCATCCTCCTACGGACCATGAACTCACTCAAGCAATTGTACACAGTGACTATGGATGTGCATTCCAGGTACAGAACTGAGGCCCATCAGGATGTGGTGGGAAGATTTAATGAAAGGTTTATTCTGTCTCTGGCCTCTTGTAAGAAGTGTCTCGTCATTGATGACCAGCTCAACATCCTGCCCATCTCCTCCCACGTTGCCACCATGGAGGCCCTGCCTCCCCAGACTCCGGATGAGAGTCTTGGTCCTTCTGATCTGGAGCTGAGGGAGTTGAAGGAGAGCTTGCAGGACACCCAGCCTGTGGGTGTGTTGGTGGACTGCTGTAAGACTCTAGACCAGGCCAAAGCTGTCTTGAAATTTATCGAGGGCATCTCTGAAAAGACCCTGAGGAGTACTGTTGCACTCACAGCTGCTCGAGGACGGGGAAAATCTGCAGCCCTGGGATTGGCGATTGCTGGGGCGGTGGCATTTGGGTACTCCAATATCTTTGTTACCTCCCCAAGCCCTGATAACCTCCATACTCTGTTTGAATTTGTATTTAAAGGATTTGATGCTCTGCAATATCAGGAACATCTGGATTATGAGATTATCCAGTCTCTAAATCCTGAATTTAACAAAGCAGTGATCAGAGTGAATGTATTTCGAGAACACAGGCAGACTATTCAGTATATACATCCTGCAGATGCTGTGAAGCTGGGCCAGGCTGAACTAGTTGTGATTGATGAAGCTGCCGCCATCCCCCTCCCCTTGGTGAAGAGCCTACTTGGCCCCTACCTTGTTTTCATGGCATCCACCATCAATGGCTATGAGGGCACTGGCCGGTCACTGTCCCTCAAGCTAATTCAGCAGCTCCGTCAACAGAGCGCCCAGAGCCAGGTCAGCACCACTGCTGAGAATAAGACCACGACGACAGCCAGATTGGCATCAGCGCGGACACTGTATGAGGTTTCCCTCCAGGAGTCAATCCGATACGCCCCTGGGGATGCAGTGGAGAAGTGGCTGAATGACTTGCTGTGCCTGGATTGCCTCAACATCACTCGGATAGTCTCAGGCTGCCCCTTGCCTGAAGCTTGTGAACTGTACTATGTTAATAGAGATACCCTCTTTTGCTACCACAAGGCCTCTGAAGTTTTCCTCCAACGGCTTATGGCCCTCTACGTGGCTTCTCACTACAAGAACTCTCCCAATGATCTCCAGATGCTCTCCGATGCACCTGCTCACCATCTCTTCTGCCTTCTGCCTCCCGTGCCCCCCACCCAGAATGCCCTTCCAGAAGTGCTTGCTGTTATCCAGGTGTGCCTTGAAGGGGAGATTTCTCGCCAGTCCATCTTGAACAGTCTGTCTCGAGGCAAGAAGGCTTCAGGGGACCTGATTCCATGGACAGTGTCAGAACAGTTCCAAGATCCAGACTTTGGTGGTCTGTCTGGTGGAAGGGTCGTTCGCATTGCTGTTCACCCAGATTATCAAGGGATGGGCTATGGCAGCCGTGCTCTGCAGCTGCTGCAGATGTACTATGAAGGCAGGTTTCCTTGTCTGGAGGAAAAGGTCCTTGAGACACCACAGGAAATTCACACCGTAAGCAGCGAGGCTGTCAGCTTGTTGGAAGAGGTCATCACTCCCCGGAAGGACCTGCCTCCTTTACTCCTCAAATTGAATGAGAGGCCTGCCGAACGCCTGGATTACCTGGGTGTTTCCTATGGCTTGACCCCCAGGCTCCTCAAGTTCTGGAAACGAGCTGGATTTGTTCCTGTTTATCTGAGACAGACCCCGAATGACCTGACCGGAGAGCACTCGTGCATCATGCTGAAGACGCTCACTGATGAGGATGAGGCTGACCAGGGAGGCTGGCTTGCAGCCTTCTGGAAAGATTTCCGACGGCGGTTCCTAGCCTTGCTCTCCTACCAGTTCAGTACCTTCTCTCCTTCCCTGGCTCTGAACATCATTCAGAACAGGAACATGGGGAAGCCAGCCCAGCCTGCCCTGAGCCGGGAGGAGCTGGAAGCACTCTTCCTCCCCTATGACCTGAAGCGGCTGGAGATGTATTCACGGAATATGGTGGACTATCACCTCATCATGGACATGATCCCGGCCATCTCTCGCATCTATTTCCTGAACCAGCTGGGGGACCTGGCCCTGTCTGCGGCTCAGTCGGCTCTTCTCTTGGGGATTGGCCTGCAGCATAAGTCTGTGGACCAGCTGGAAAAGGAGATTGAGCTGCCCTCGGGCCAGTTGATGGGACTTTTCAACCGGATCATCCGCAAAGTTGTGAAGCTATTTAATGAAGTTCAGGAAAAGGCCATTGAGGAGCAGATGGTGGCAGCGAAGGATGTGGTCATGGAGCCCACGATGAAGACCCTCAGTGACGACCTAGATGAAGCAGCAAAGGAATTTCAGGAGAAACACAAGAAGGAAGTAGGGAAGCTGAAGAGCATGGACCTCTCTGAATACATAATCCGTGGGGACGATGAAGAGTGGAATGAAGTTTTGAACAAAGCTGGGCCGAACGCCTCGATCATCAGCCTGAAAAGTGACAAGAAAAGGAAGTTAGAGGCCAAACAAGAACCCAAACAGAGCAAGAAGTTGAAGAACAGAGAGACAAAGAACAAAAAAGATATGAAACTGAAGCGGAAGAAATAG |
| --- | --- |
| OE-COL5A1 | ATGGACGTCCATACCCGCTGGAAAGCGCGCAGCGCGCTCCGCCCGGGCGCCCCGCTGCTGCCCCCGCTGCTGCTGCTGCTGCTGTGGGCGCCGCCTCCGAGCCGCGCAGCTCAGCCAGCAGATCTCCTGAAGGTTCTAGATTTTCACAACTTGCCTGATGGAATAACAAAGACAACAGGCTTTTGCGCCACGCGGCGATCTTCCAAAGGCCCGGATGTCGCTTACAGAGTCACCAAAGACGCGCAGCTCAGCGCACCCACCAAGCAGCTGTACCCTGCGTCTGCATTTCCCGAGGACTTCTCCATCCTAACAACTGTGAAAGCCAAGAAAGGCAGCCAGGCCTTCCTGGTCTCCATCTACAACGAGCAGGGTATCCAGCAGATTGGGCTGGAGCTGGGCCGCTCTCCCGTCTTCCTCTACGAGGACCACACGGGGAAGCCTGGCCCGGAAGACTACCCCCTCTTCCGGGGCATCAACCTGTCAGATGGCAAGTGGCACAGAATTGCTCTCAGCGTCCACAAGAAAAATGTCACCTTGATCCTCGACTGTAAAAAGAAGACCACCAAATTCCTCGACCGCAGCGACCACCCCATGATCGACATCAATGGCATCATCGTGTTTGGCACCCGGATCCTGGATGAGGAGGTGTTTGAGGGTGACATCCAGCAGCTGCTCTTTGTCTCGGACCACCGGGCAGCTTATGATTACTGTGAGCACTACAGCCCTGACTGTGACACCGCAGTACCTGACACCCCACAGTCGCAGGACCCCAATCCAGATGAATATTACACGGAAGGAGACGGCGAGGGTGAGACCTATTACTACGAATACCCCTACTACGAAGACCCCGAAGACCTAGGGAAGGAGCCCACCCCCAGCAAGAAGCCCGTGGAAGCTGCCAAAGAAACCACAGAGGTCCCCGAGGAGCTGACCCCGACCCCCACGGAAGCTGCTCCCATGCCTGAAACCAGTGAAGGGGCTGGGAAGGAAGAGGACGTCGGCATCGGGGACTATGACTACGTGCCCAGTGAGGACTACTACACGCCCTCACCGTATGATGACCTCACCTATGGCGAGGGGGAGGAGAACCCCGACCAGCCCACAGACCCAGGCGCTGGGGCCGAAATTCCCACCAGCACCGCCGACACCTCCAACTCCTCCAATCCAGCTCCGCCTCCAGGGGAAGGTGCGGATGACTTGGAGGGGGAGTTCACTGAGGAAACGATCCGGAACCTTGACGAGAACTACTACGACCCCTACTACGACCCCACCAGCTCCCCGTCGGAGATCGGGCCGGGAATGCCGGCGAACCAGGATACCATCTATGAAGGGATTGGAGGACCTCGGGGCGAGAAAGGCCAAAAGGGAGAACCAGCGATTATCGAGCCGGGCATGCTCATCGAGGGCCCGCCTGGCCCAGAAGGCCCCGCGGGTCTTCCCGGACCTCCAGGAACCATGGGTCCCACTGGCCAAGTCGGGGACCCTGGAGAAAGGGGCCCCCCTGGACGCCCAGGCCTTCCTGGGGCCGATGGCCTGCCCGGTCCTCCAGGAACCATGCTCATGCTGCCCTTCCGGTTTGGAGGTGGCGGCGATGCGGGCTCCAAAGGCCCCATGGTCTCAGCCCAGGAGTCCCAGGCGCAAGCCATTCTCCAGCAGGCCAGGTTGGCACTGAGGGGACCAGCTGGCCCGATGGGTCTCACAGGGAGACCTGGCCCTGTGGGTCCCCCTGGGAGCGGAGGTTTGAAGGGCGAGCCGGGAGACGTGGGGCCTCAGGGTCCTCGAGGTGTGCAAGGCCCGCCTGGTCCGGCCGGGAAGCCCGGAAGACGGGGTCGGGCTGGGAGTGATGGAGCCAGAGGAATGCCTGGACAAACTGGCCCCAAGGGTGACCGGGGTTTCGACGGCCTGGCTGGGTTGCCAGGCGAGAAGGGCCACAGGGGTGACCCTGGTCCTTCCGGCCCACCAGGACCTCCGGGAGACGATGGAGAAAGGGGTGACGACGGAGAAGTTGGGCCCAGGGGGCTGCCTGGGGAGCCCGGGCCACGTGGTCTGCTTGGGCCGAAGGGGCCCCCAGGTCCTCCCGGACCTCCCGGTGTCACGGGTATGGACGGCCAGCCGGGGCCAAAAGGAAATGTGGGTCCCCAGGGAGAGCCTGGCCCCCCAGGACAGCAGGGTAATCCAGGCGCCCAGGGTCTTCCAGGCCCCCAGGGTGCAATTGGTCCTCCAGGAGAAAAGGGTCCCTTGGGGAAACCAGGCCTTCCAGGAATGCCCGGTGCTGACGGACCCCCGGGACACCCTGGCAAAGAAGGCCCTCCAGGAGAGAAAGGAGGTCAGGGTCCACCTGGCCCCCAGGGTCCGATTGGCTACCCAGGTCCTCGAGGAGTCAAGGGGGCCGATGGCATCCGTGGTCTGAAGGGCACAAAGGGCGAGAAGGGTGAAGACGGCTTTCCTGGGTTTAAAGGAGACATGGGCATCAAGGGTGATCGGGGGGAGATCGGCCCACCCGGTCCCAGGGGAGAAGATGGCCCTGAAGGCCCAAAGGGTCGCGGAGGTCCCAATGGTGACCCCGGTCCTCTGGGACCCCCTGGGGAGAAGGGAAAACTCGGAGTCCCAGGGTTACCAGGGTATCCAGGAAGACAAGGACCAAAGGGCTCTATTGGATTCCCTGGATTTCCTGGCGCCAATGGAGAGAAGGGCGGCAGGGGGACCCCTGGAAAGCCAGGACCGCGGGGGCAGCGAGGCCCAACGGGTCCGAGGGGTGAAAGAGGCCCCCGGGGCATCACTGGGAAGCCTGGCCCCAAGGGCAACTCCGGAGGTGACGGCCCAGCTGGCCCTCCTGGTGAACGGGGACCCAATGGACCCCAAGGACCCACAGGATTTCCTGGACCAAAGGGCCCCCCTGGCCCTCCAGGCAAGGATGGACTCCCAGGACACCCTGGACAGAGAGGCGAGACTGGTTTCCAAGGCAAGACCGGCCCTCCAGGCCCCCCCGGCGTGGTCGGCCCTCAGGGTCCCACGGGAGAAACGGGCCCAATGGGTGAGCGTGGCCACCCTGGGCCCCCTGGACCCCCCGGTGAACAGGGGCTTCCGGGCCTTGCTGGAAAAGAAGGGACGAAGGGTGACCCAGGCCCTGCAGGCCTCCCTGGGAAAGATGGCCCTCCAGGATTACGTGGTTTCCCTGGGGACCGAGGGCTTCCTGGTCCAGTGGGAGCTCTTGGACTGAAAGGCAATGAAGGGCCCCCTGGCCCACCAGGCCCTGCGGGATCTCCAGGGGAGAGAGGTCCAGCTGGAGCCGCTGGGCCCATCGGAATTCCAGGGAGACCTGGGCCCCAGGGACCCCCAGGGCCGGCAGGAGAGAAAGGGGCTCCTGGCGAGAAAGGCCCACAAGGCCCAGCTGGCCGAGACGGTCTCCAGGGGCCTGTGGGGCTCCCGGGTCCAGCTGGCCCTGTGGGTCCCCCTGGAGAAGACGGAGATAAGGGAGAGATCGGGGAGCCGGGGCAGAAAGGAAGCAAGGGGGACAAAGGAGAACAGGGTCCTCCTGGGCCTACAGGTCCTCAAGGCCCCATCGGACAGCCAGGCCCCTCTGGAGCTGACGGCGAGCCGGGGCCTCGGGGCCAGCAGGGCCTTTTCGGGCAGAAAGGTGATGAAGGTCCCAGAGGCTTTCCTGGACCCCCTGGGCCAGTGGGGCTGCAGGGTTTGCCAGGACCTCCAGGCGAGAAGGGTGAGACAGGAGACGTGGGCCAGATGGGCCCCCCGGGTCCCCCTGGCCCCCGAGGACCCTCCGGAGCTCCAGGTGCTGATGGCCCACAAGGTCCCCCAGGTGGAATAGGAAACCCTGGTGCAGTGGGAGAGAAGGGCGAGCCTGGCGAAGCAGGTGAGCCTGGCCTTCCGGGAGAAGGCGGCCCCCCGGGACCCAAAGGAGAAAGGGGAGAGAAGGGCGAGTCAGGCCCTTCAGGTGCTGCCGGACCCCCTGGACCCAAAGGCCCTCCCGGAGATGATGGTCCCAAAGGCAGCCCTGGCCCAGTGGGTTTTCCTGGAGATCCTGGCCCCCCCGGAGAGCCTGGCCCCGCGGGTCAAGATGGTCCCCCTGGTGACAAAGGAGATGATGGTGAACCCGGGCAGACGGGATCCCCCGGCCCTACTGGTGAACCAGGTCCATCGGGGCCTCCAGGAAAAAGGGGTCCCCCAGGCCCCGCAGGCCCCGAAGGCAGACAGGGAGAGAAAGGGGCCAAGGGAGAAGCCGGCTTGGAAGGCCCTCCTGGGAAGACTGGCCCCATCGGCCCCCAGGGGGCCCCTGGGAAGCCCGGACCGGATGGCCTTCGAGGGATCCCTGGCCCTGTGGGAGAACAAGGTCTCCCAGGATCCCCAGGCCCGGACGGTCCCCCCGGCCCCATGGGTCCCCCAGGACTTCCCGGCCTCAAAGGAGATTCTGGTCCCAAAGGTGAAAAGGGTCATCCAGGCCTGATCGGGCTCATCGGTCCTCCGGGTGAACAGGGTGAGAAGGGCGACCGTGGTCTCCCTGGCCCCCAGGGCTCCTCCGGTCCTAAGGGAGAACAGGGTATCACTGGTCCTTCTGGCCCGATTGGGCCTCCTGGGCCCCCTGGCCTGCCGGGTCCGCCTGGTCCAAAAGGTGCTAAGGGCTCCTCGGGTCCAACTGGCCCGAAGGGTGAGGCAGGCCACCCAGGACCCCCAGGCCCCCCGGGCCCCCCGGGAGAGGTCATCCAGCCCCTGCCAATCCAGGCATCCAGGACGCGGCGGAACATCGACGCCAGCCAGCTGCTGGACGACGGGAATGGCGAGAACTACGTGGACTACGCGGACGGCATGGAAGAGATCTTCGGCTCTCTCAACTCTCTGAAGCTGGAGATTGAGCAGATGAAACGGCCCCTGGGCACGCAGCAGAACCCCGCCCGCACCTGCAAGGACCTGCAGCTCTGCCACCCCGACTTCCCAGATGGTGAATACTGGGTCGATCCTAACCAAGGATGCTCCAGGGATTCCTTCAAGGTTTACTGCAACTTCACAGCCGGGGGGTCGACATGCGTCTTCCCTGACAAGAAGTCCGAAGGGAGTAAAATGGCCCGCTGGCCCAAAGAGCAGCCTTCCACCTGGTATAGTCAGTACAAGCGGGGGTCCCTGCTCTCCTATGTGGACGCCGAGGGCAACCCTGTGGGTGTGGTACAGATGACCTTCCTGCGGCTGCTGAGCGCCTCTGCCCACCAGAACGTCACCTACCACTGCTACCAGTCAGTGGCCTGGCAGGACGCAGCCACGGGCAGCTACGACAAGGCCCTCCGCTTCCTGGGCTCCAACGACGAGGAGATGTCCTATGACAACAACCCCTACATCCGCGCCCTGGTGGACGGCTGTGCTACCAAGAAAGGCTACCAGAAGACGGTTCTGGAGATCGACACCCCCAAAGTGGAGCAGGTGCCCATCGTGGACATCATGTTCAATGACTTCGGTGAAGCGTCACAGAAATTTGGATTTGAAGTGGGGCCGGCTTGCTTCATGGGCTAG |
| WT-3’UTR sequence | GAGCCGCC GAGCCCGGGC TCCCGAGAGC AACCTCGTGA CCTCAGCATG CCATTCGTTC GTGAGTGTCC CGTGCACGTC CTGACCCTGG ACAGTGAAGG CTTCTCCCTC CCCTCCCACC TGACTTCATC TACGCCTCGG CACCACGGGG TGTGGGACCC CAGCCCGGAG AGAACAGAGG GAAGGAGCCG CGCCCCCACC TGGAGCTGAA TCACATGACC TAGCTGCACC CCAGCGCCTG GGCCCGCCCC ACGCTCTGTC CACACCCACG CGCCCCGGGA GCGGGGCCAT GCCTCCAGCC CCCCAGCTCG CCCGACCCAT CCTGTTCGTG AATAGGTCTC AGGGGTTGGG GGAGGGACTG CCAGATTTGG ACACTATATT TTTTTCTAAA TTCAACTTGA AGATGTGTAT TTCCCCTGAC CTTCAAAAAA TGTTCCAAGG TAAGCCTCGT AAAGGTCATC CCACCATCAC CAAAGCCTCC GTTTTTAACA ACCTCCAACA CGATCCATTT AGAGGCCAAA TGTCATTCTG CAGGTGCCTT CCCGATGGAT TAAAGGTGCT TATGTTTTTG TGAGTTTTAA GTAAATATTT GTATTGTATT GTTATAAATG TTAAGTGTGC CTGGCTTTCA ATCATGCACG GAAACCCAGT CTCAGTCCCA CGGACAGAAT GGGCGAGGCA TGGATTCTGG GTTGCAGTAC CGTTCTGATT AGAAATAGGA AGTCTCCCCA CCCCCGCCCT GGCCAAGAAC GTGCAATAAA TTGGAAGTTT GCCCCGGGGC AGCAAGAATT TATGCTGCCA TTGAAAAGCA GGTACCAGTG CCCCTTTTCA GACAGTTTTT GATTCGCTCT AGACTTTTTT TTTTTTTAAT AGGGAAAAAA TTTGATAATT TTCTTTTTTC TACATGCACT TAAGACTAAA ACACAGGTTT GGATTAATTT TATTTGCTTC CTTTTTCCGC TTTTCTTCCC GCAGAGCCTG ATGGGAGAAT GTCCAGGGCA GGGAAACCAC ATTTTTTGTA GGTGATAACT CAATGAAAAT TGGTGCTTAT TTTTTACACT TCTCTCTTGT GGCTCTCTTG TGGTGCTATC TATCTGTTTT AAGGTCTCCT TGAAGGCGCA CTGGGGACCC TGGCCATGCC TCGTTCTCCC TGCTTTCTTT ATCCTGTTAT TGCCTCCACA GTCTGTTGCC AAGGACTCTA AGATCAATGC ACGTCACTTT CCTTTCCACT GGGCAGGATA GCCAAGCACA CTCCCTCCTG CGCTCTCCCG CCCCGGTGCG TCCACTCCCG AGGGCTGTTA TGAGGACTGG GTTGTGCCTA CTTGATTTGA AAACACACAC AAGCAATAAA AAGCCTCTTC CTGCATTGTC TGTGGTGTGA CCATAGCAGA TTATATTTGG TTCCTGAATG TTTGTGGTGC TAATTTCTGT GTTTGTTCCA AGCCGTTCAG TCATGCCATG CGCTGCCTCG GTAGATGGAG TAATGTACAA TGAACTCCAT GAGTCTCTCC AGGGCTGCCT GCAGCACGTC TTTTCCAAGT AGCCTATTTG GATTCCCATC TCAAATGTCC TGGATGCGAG CGTCAGCGGC TCCAGAGCTC GGGGCGGGTG AGGTCCCCTT TGGGGAACCC TTTCCTGGCC ATCGAGGTCG GGGGGCTGCC GTCTGTGGGC AGGAGGACCC GAGGGGCAGC CAGGAAAGGC GATCTCTTCA CTGTGAAAAG TTGCCCGGGT GCAGCGCCTT TTCCTTCTAC CATGGGAAAT GCAGGCTGGG CCCTTGGGGT GAGCCTGCGG GGCTCTGGTG CTGTCCCCGA CCCCCACCAC CACCAGAATG CAGTTCCAGC TTAGGAAGCC ACAAACAAGC CACCCAGGAG GAACAAAACA CCGCCAGCGT GGATTTTCCA AATTTCCCTG GAAAGTAAGT CTCGCTCTTG CCAAAGAAAA GTCTGGCTTG GAGAGTCTCT GGAGCCCAGG ATGCCAGCAT GTGCCAATGA CTGTCACCTT CATCTCTTCA AAAGAAAAGC CATAGCCGAG GACTGTCCCG CGACCCCCGT GGACTGCGTC TAGGTCATGT GATTCTGTTT TCATTTCTCA TCCCATCCAA TTTGTCCTTT TCTCCTGTCA TTTTCTTCCT CTGTGGTCCC TTCAAAGTTG TTATAATTTG TACTGAACTT CAAAATGTGT CCCGTTCTCC CCAGACCACT CTAGCCACAG TATATTGCAA TAAAATTACT TCTTATATTT GCAGAAATTC TTTTGGTGTA ATTTTATTTT TTCCTCTCAA TATATATAAT TGGACAAACG CTGGCAAAAA GAAAAAAATG GTAAGCAAAA AACCCAAGAT AAAGTTTCGA GGACATCAGG CCTTTTGAAA TACAATGTCA AATGACACAT TGTACGGTTT CAAAAAATCC GCTAGACATG TCATAAGTTT TAACTGTAAT GCCCAGGAAA GGATATCTTA AAATATTCTA AACTTGTGTA ACAAAGGAAT AATTAACTGT AATAGTTTTT CAATAAATCG AGTTGGGTGT TTCCACCGTA AA |
| MUT-3’UTR sequence | GAGCCGCC GAGCCCGGGC TCCCGAGAGC AACCTCGTGA CCTCAGCATG CCATTCGTTC GTGAGTGTCC CGTGGAGGTC CTGACCCTGG ACAGTGAAGG CTTCTCCCTC CCCTCCGAGC TGACTTCATC TACGCCTCGG GAGGAGGGGG TGTGGGACCC CAGCCCGGAG AGAACAGAGG GAAGGAGCCG CGCCCCGAGC TGGAGCTGAA TGAGATGACC TAGCTGGAGC CCAGCGCCTG GGCCCGCCCC ACGCTCTGTC GAGACCGAGG CGCCCCGGGA GCGGGGCCAT GCCTCCAGCC CCCCAGCTCG CCCGACCCAT CCTGTTCGTG AATAGGTCTC AGGGGTTGGG GGAGGGACTG CCAGATTTGG AGAGTATATT TTTTTCTAAA TTCAACTTGA AGATGTGTAT TTCCCCTGAC CTTCAAAAAA TGTTCCAAGG TAAGCCTCGT AAAGGTCATC CGAGCATGAG CAAAGCCTCC GTTTTTAACA ACCTCCAACA CGATCCATTT AGAGGCCAAA TGTCATTCTG CAGGTGCCTT CCCGATGGAT TAAAGGTGCT TATGTTTTTG TGAGTTTTAA GTAAATATTT GTATTGTATT GTTATAAATG TTAAGTGTGC CTGGCTTTCA ATCATGGAGG GAAACCCAGT CTCAGTCCCA CGGACAGAAT GGGCGAGGCA TGGATTCTGG GTTGCAGTAC CGTTCTGATT AGAAATAGGA AGTCTCCCCA CCCCCGCCCT GGCCAAGAAC GTGCAATAAA TTGGAAGTTT GCCCCGGGGC AGCAAGAATT TATGCTGCCA TTGAAAAGCA GGTACCAGTG CCCCTTTTCA GACAGTTTTT GATTCGCTCT AGACTTTTTT TTTTTTTAAT AGGGAAAAAA TTTGATAATT TTCTTTTTTC TACATGGAGT TAAGACTAAA AGAGAGGTTT GGATTAATTT TATTTGCTTC CTTTTTCCGC TTTTCTTCCC GCAGAGCCTG ATGGGAGAAT GTCCAGGGCA GGGAAACGAG ATTTTTTGTA GGTGATAACT CAATGAAAAT TGGTGCTTAT TTTTTAGAGT TCTCTCTTGT GGCTCTCTTG TGGTGCTATC TATCTGTTTT AAGGTCTCCT TGAAGGCGCA CTGGGGACCC TGGCCATGCC TCGTTCTCCC TGCTTTCTTT ATCCTGTTAT TGCCTCGAGA GTCTGTTGCC AAGGACTCTA AGATCAATGC ACGTGAGTTT CCTTTCGAGT GGGCAGGATA GCCAAGGAGA CTCCCTCCTG CGCTCTCCCG CCCCGGTGCG TCGAGTCCCG AGGGCTGTTA TGAGGACTGG GTTGTGCCTA CTTGATTTGA AAAGAGAGAG AAGCAATAAA AAGCCTCTTC CTGCATTGTC TGTGGTGTGA CCATAGCAGA TTATATTTGG TTCCTGAATG TTTGTGGTGC TAATTTCTGT GTTTGTTCCA AGCCGTTCAG TCATGCCATG CGCTGCCTCG GTAGATGGAG TAATGTACAA TGAACTCCAT GAGTCTCTCC AGGGCTGCCT GCAGGAGGTC TTTTCCAAGT AGCCTATTTG GATTCCCATC TCAAATGTCC TGGATGCGAG CGTCAGCGGC TCCAGAGCTC GGGGCGGGTG AGGTCCCCTT TGGGGAACCC TTTCCTGGCC ATCGAGGTCG GGGGGCTGCC GTCTGTGGGC AGGAGGACCC GAGGGGCAGC CAGGAAAGGC GATCTCTTCA CTGTGAAAAG TTGCCCGGGT GCAGCGCCTT TTCCTTCTAC CATGGGAAAT GCAGGCTGGG CCCTTGGGGT GAGCCTGCGG GGCTCTGGTG CTGTCCCCGA CCCCGAGGAG GAGCAGAATG CAGTTCCAGC TTAGGAAGCC ACAAACAAGC GAGCCAGGAG GAACAAAACA CCGCCAGCGT GGATTTTCCA AATTTCCCTG GAAAGTAAGT CTCGCTCTTG CCAAAGAAAA GTCTGGCTTG GAGAGTCTCT GGAGCCCAGG ATGCCAGCAT GTGCCAATGA CTGTGAGCTT CATCTCTTCA AAAGAAAAGC CATAGCCGAG GACTGTCCCG CGACCCCCGT GGACTGCGTC TAGGTCATGT GATTCTGTTT TCATTTCTCA TCCCATCCAA TTTGTCCTTT TCTCCTGTCA TTTTCTTCCT CTGTGGTCCC TTCAAAGTTG TTATAATTTG TACTGAACTT CAAAATGTGT CCCGTTCTCC CCAGACGAGT CTAGCGAGAG TATATTGCAA TAAAATTACT TCTTATATTT GCAGAAATTC TTTTGGTGTA ATTTTATTTT TTCCTCTCAA TATATATAAT TGGACAAACG CTGGCAAAAA GAAAAAAATG GTAAGCAAAA AACCCAAGAT AAAGTTTCGA GGACATCAGG CCTTTTGAAA TACAATGTCA AATGAGAGAT TGTACGGTTT CAAAAAATCC GCTAGACATG TCATAAGTTT TAACTGTAAT GCCCAGGAAA GGATATCTTA AAATATTCTA AACTTGTGTA ACAAAGGAAT AATTAACTGT AATAGTTTTT CAATAAATCG AGTTGGGTGT TTCGAGCGTA AA |

Data S1. (separate file)

The original data of Fig. 1k


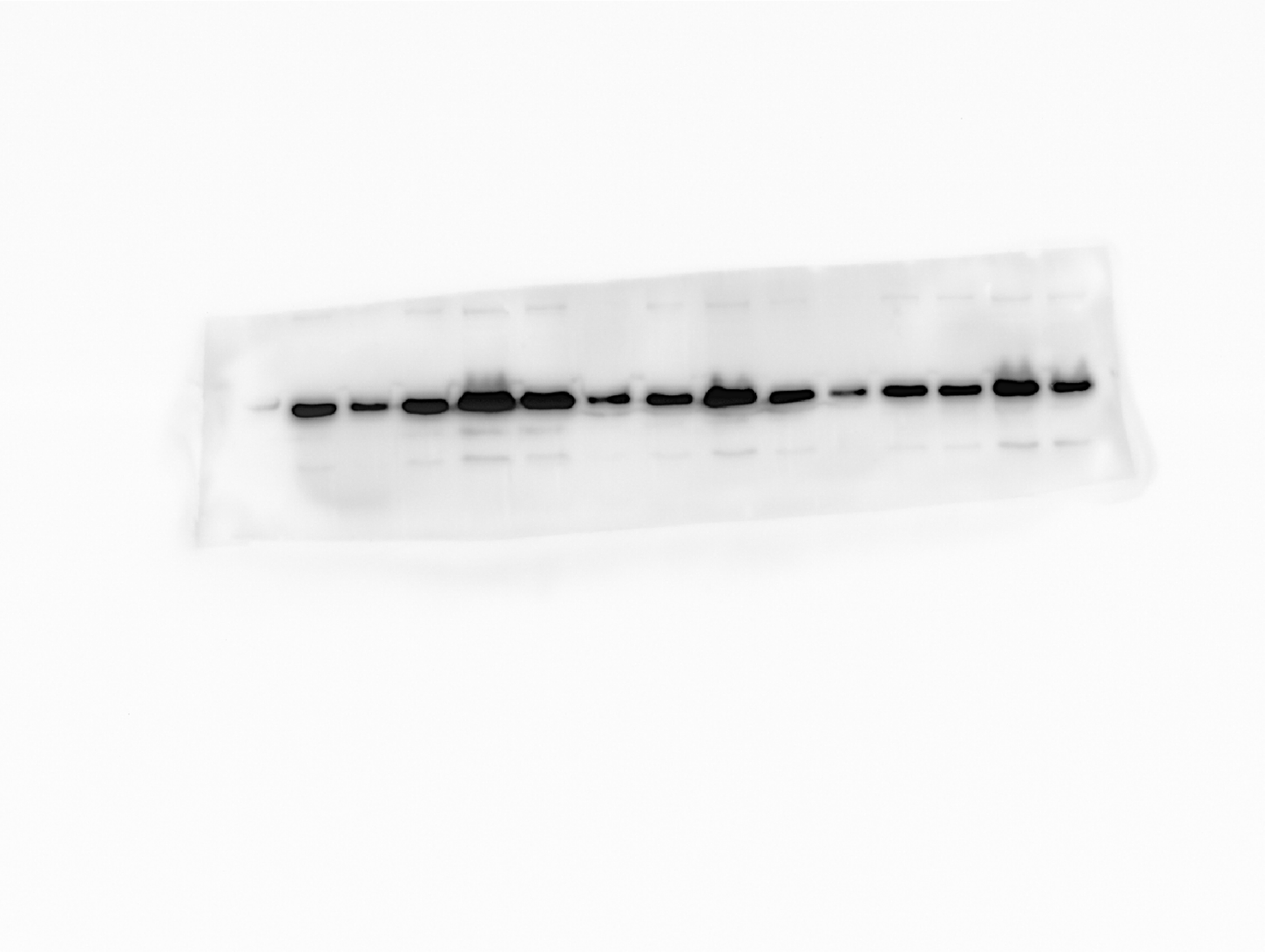


The original data of MGC-803 COL5A1 (patch 1-4), SGC-7901 COL5A1 (patch 5-8)


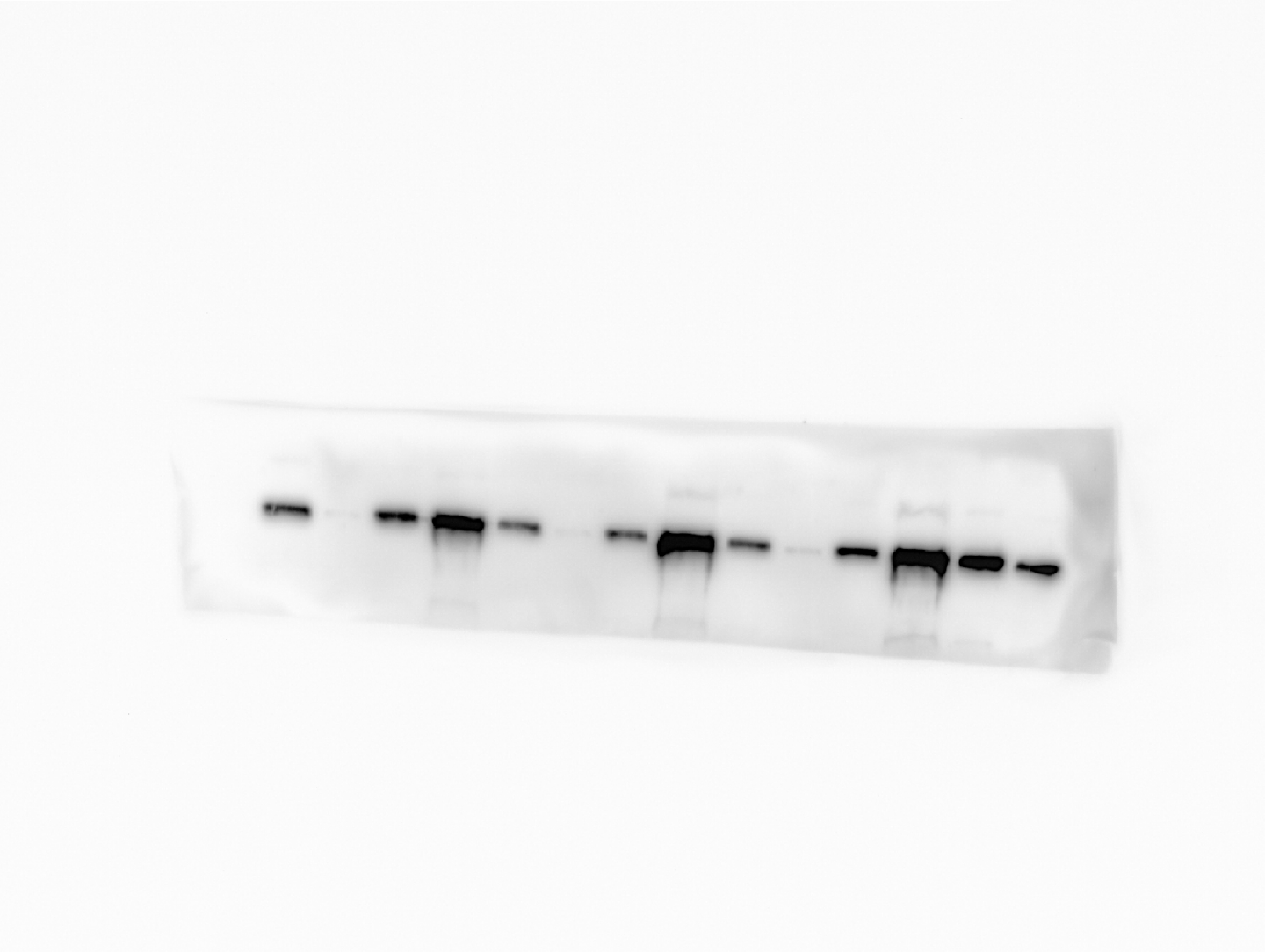


The original data of MGC-803 NAT10 (patch 1-4), SGC-7901 NAT10 (patch 5-8)


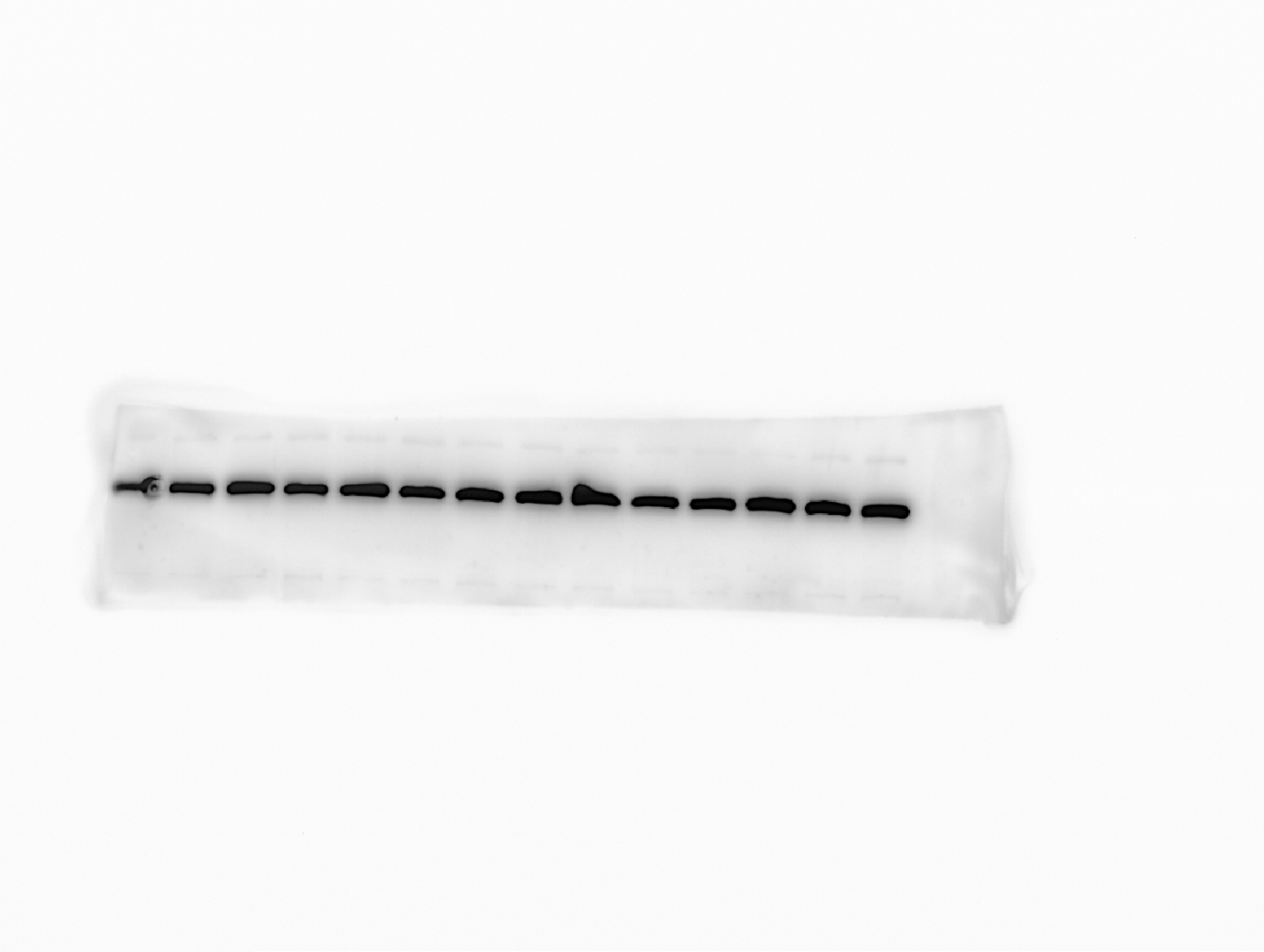


The original data of MGC-803 GAPDH (patch 2-5), SGC-7901 GAPDH ­­­­­(patch11-14)
